# Associations between perfluoroalkyl substances and serum lipids in a Swedish adult population with contaminated drinking water

**DOI:** 10.1186/s12940-020-00588-9

**Published:** 2020-03-14

**Authors:** Ying Li, Lars Barregard, Yiyi Xu, Kristin Scott, Daniela Pineda, Christian H. Lindh, Kristina Jakobsson, Tony Fletcher

**Affiliations:** 1 School of Public Health and Community Medicine, Institute of Medicine, University of Gothenburg, Box 414, SE, 405 30 Gothenburg, Sweden; 2 Occupational and Environmental Medicine, Sahlgrenska University Hospital, Gothenburg, Sweden; 3 Division of Occupational and Environmental Medicine, Department of Laboratory Medicine, Lund University, Lund, Sweden; 4 London School of Hygiene and Tropical Medicine, London, UK

**Keywords:** Serum lipids, Cholesterol, Perfluoroalkyl substances (PFAS), Perfluorooctane sulfonic acid (PFOS), Perfluorooctanoic acid (PFOA), Perfluorohexane sulfonic acid (PFHxS)

## Abstract

**Background:**

Exposures to perfluoroalkyl substances (PFAS) have shown positive associations with serum lipids in previous studies. While many studies on lipids investigated associations with perfluorooctane sulfonic acid (PFOS) and perfluorooctanoic acid (PFOA), there are only a few studies regarding other PFAS, such as perfluorohexane sulfonic acid (PFHxS). The purpose of the current study is to investigate if associations with serum lipids were present, not only for serum PFOS and PFOA, but also for PFHxS, and if the associations with PFAS remained also in a comparison based only on residency in areas with contrasting exposure to PFAS.

**Methods:**

1945 adults aged 20–60 were included from Ronneby, Sweden, a municipality where one out of two waterworks had been heavily contaminated from aqueous fire-fighting foams, and from a nearby control area. The exposure was categorized based on either been living in areas with contrasting PFAS exposure or based on the actual serum PFAS measurements. Regression analyses of serum lipids were fitted against serum PFAS levels, percentile groups, smooth splines and between exposed and reference areas, adjusting for age, sex and BMI.

**Results:**

Drinking water contamination caused high serum levels of PFOS (median 157 ng/ml) and PFHxS (median 136 ng/ml) and PFOA (median 8.6 ng/ml). These serum PFAS levels in the exposed groups were 5 to 100-fold higher than in the controls. In this population with mixed PFAS exposure, predominantly PFOS and PFHxS, PFAS exposure were positively associated with serum lipids. This was observed both when quantifying exposure as contrast between exposed and controls, and in terms of serum PFAS. Due to high correlations between each PFAS, we cannot separate them.

**Conclusions:**

In conclusion, the present study provides further evidence of a causal association between PFAS and serum lipids, especially for PFHxS.

## Background

Perfluoroalkyl substances (PFAS) are persistent substances which have been produced and used in industry and in household products due to their surfactant and repellant properties [1]. They have also been used as surfactants in fire-fighting foam [2]. PFAS are ubiquitous in the environment and sources of exposure to PFAS in the general population may include food, drinking water, house dust, air, placental transfer and breast milk for infants [3, 4]. Contaminated drinking water can lead to substantial PFAS exposure, as in the US C8 cohort [5] and in a Swedish cohort [6].

Exposure to PFAS has been associated with a number of health effects in humans [7]. Positive associations between PFAS and serum lipids, in particular total cholesterol and low-density lipoprotein (LDL) cholesterol have been shown in more than 20 studies; for a summary see a recent review by the European Food Safety Authority [7]. While many studies investigated associations with perfluorooctane sulfonic acid (PFOS) and perfluorooctanoic acid (PFOA), there are only a few studies regarding other PFAS, such as perfluorohexane sulfonic acid (PFHxS) [8–11].

Regarding causality of the associations between PFAS and serum lipids it should be noted that most of the studies were cross-sectional, although an association was also found in some longitudinal studies, for example a study evaluating the change of serum lipids versus the change of serum PFOS and PFOA over a 4-year period [12], a study on serum lipids versus modelled exposure to PFOA from drinking water [13], and a study on examining PFAS-lipids relationship in pre-diabetic adults over 15 years [14]. The issue of potential confounding by dietary factors, or reverse causality (by binding of PFAS to lipoproteins), was discussed extensively by EFSA [7]. Moreover, confounding by variability in intestinal reabsorption of PFAS and bile acids, which in turn is associated with serum cholesterol, remains an important issue when exposure to PFAS is assessed by serum PFAS levels [15, 16]. PFAS are excreted in bile, but reabsorbed in the intestine to a high extent, and relatively high reabsorption should be associated with higher steady state PFAS levels in serum. This specific source of potential confounding may be present when exposure is based on serum levels of PFAS but not when it is based on estimation of intake due to residence in a water district with PFAS-contaminated drinking water.

The aim of the present study in Swedish adults exposed to PFAS from contaminated drinking water, was to investigate associations between exposure to PFAS and serum lipids. In particular, we examined if associations were present, not only for serum PFOS and PFOA, but also for PFHxS, and if the associations with PFAS were also present in a comparison based only on residency in areas with contrasting exposure to PFAS.

## Methods

### Study population

As described in detail in Li [6], in 2013, it was noted that one of the two waterworks in the municipality of Ronneby in Southern Sweden was contaminated with PFAS. This waterworks provided drinking water to about 10,000 people in part of the municipality. It was soon confirmed that the use of aqueous film-forming foam (AFFF) at the nearby military airport localized within the aquifer area was the source of this contamination, which likely had started in the mid-1980s. The contaminated waterworks was closed in December 2013, and clean water was provided by the other waterworks. Levels of several PFAS in contaminated drinking water were clearly elevated: PFOS, 8000 ng/L, PFHxS, 1700 ng/L, perfluorohexanoic acid (PFHxA), 320 ng/L, perfluorobutane sulfonic acid (PFBS), 130 ng/L and PFOA, 100 ng/L. In contrast, the other waterworks in the municipality had provided water with PFAS levels well below Swedish guidance values (Supplementary Table S1).

Extensive biomonitoring in the municipality started in June 2014, approximately 6 months after end of the exposure, by open invitation and free of cost. Inhabitants living and working in the areas of the contaminated as well as the uncontaminated waterworks were invited. Considerable efforts were made to recruit persons with little exposure to the contaminated water in order to ensure a broad range of serum PFAS levels for further research on health effects. The open samplings ended in November 2015. A control group from a nearby municipality (Karlshamn) was also examined in 2016. Information and invitations about the open samplings in Ronneby and Karlshamn were disseminated through the municipal webpages and advertisements in the main local newspaper, and other local channels.

From the Ronneby and Karlshamn populations, in total 2791 adults took part in the open samplings. For 2601 out of them with full information about sex, age and body mass index (BMI), serum PFAS levels and blood lipids were available as well as yearly residential addresses from 1985 to 2013. Of these, 1945 participants were aged 20 to 60 years and are included in present study. The self-reported residence addresses were used to allocate the participants into three exposure groups:

- Control: comprising all participants from Karlshamn who had never been exposed to PFAS from contaminated water at their residential addresses (*n* = 130).
- Recently exposed: including the participants who lived in the contaminated area in Ronneby any time between 2005 to 2013 (*n* = 1160). We estimate that PFAS levels in the drinking water have accumulated since contamination started in the mid-1980s and so were lowest during the early years and highest towards the end. Here, the period 2005 to 2013 was set as an arbitrary interval capturing the higher exposures vis-a-vis earlier years.
- Non-recent/uncertain exposure (*n* = 655). This group included the participants from Ronneby who had ever lived in the contaminated waterworks distribution area in Ronneby before 2005 but not after, and the participants who had lived in the non-contaminated waterworks area in Ronneby at any time between 1985 to 2013. These participants were not drinking contaminated water at their residence but may have been exposed visiting the contaminated area, for example at work or at school.

### Chemical analysis

Venous non-fasting blood samples were taken in 5 ml red Becton Dickinson (BD, Belliver Industrial Estate, Plymouth. UK) vacutainer blood collection tubes without gel. They were left at room temperature for 30 min before centrifuged at 3000 x g for 10 min and aliquoted into cryotubes, and transported to the laboratory on dry ice. The aliquots were stored at − 80 °C until thawed, vortexed, and again aliquoted by manual pipetting for subsequent serum analyses.

Analyses of PFAS were carried out at Division of Occupational and Environmental Medicine, Lund University, using liquid chromatography coupled to tandem mass spectrometry (LC-MS/MS) and determined as the total, non-isomer specific compounds ([17]; Supplementary material 2). Briefly, proteins in the serum samples were precipitated using acetonitrile by vigorous shaking for 30 min. The samples were then centrifuged, and an aliquot of the supernatant was analyzed using a LC (UFLCXR, SHIMADZU Corporation, Kyoto, Japan) connected to the MS/MS (QTRAP 5500, AB Sciex, Foster City, CA, USA).

Serum samples from populations expected to have high exposure to PFAS were prepared using aliquots of 25 μl serum added with 75 μl of water and isotopically labeled internal standards for all compounds and 1 μl of the samples were analyzed using LC-MS/MS. If lower levels were expected, aliquots of 100 μl serum were used and 4 μl of the samples were analyzed using LC-MS/MS. In all sample batches, chemical blanks and three quality control (QC) samples, prepared in-house from serum spiked with different PFCs were included. For further details see Supplementary material 2. The laboratory participates in the Interlaboratory Comparison Investigations (ICI)/External Quality Assurance Schemes (EQUAS) exercises for the analysis of PFOS, PFHxS and PFOA, and are approved in the Human Biomonitoring for Europe (HBM4EU) project (https://www.hbm4eu.eu/). In addition, the analyses of PFOS and PFOA are part of a quality control program between analytical laboratories coordinated by University of Erlangen-Nuremberg, Germany. The sum of the 3 PFAS was calculated as the molar-weight adjusted sum of PFOS, PFHxS and PFOA (nmol/ml).

Total cholesterol, LDL-Cholesterol, HDL-Cholesterol and triglycerides in serum were analyzed at the Department of Clinical Chemistry of the Skåne University Hospital in Lund, Sweden, on a Cobas 8000 analyzer (Roche Diagnostics, Basel, Switzerland). The laboratory is accredited according to ISO 15189.

### Statistical analysis

Analyses were limited to participants aged 20 to 60 years. The teenagers below age 20 years were excluded due to the limited number of participants. Those aged over 60 years were excluded since no adults above 60 years of age were recruited from the control area. Also, lipid-lowering medication, affecting cholesterol levels, is more common in the elderly and data on medications were not available.

Analyses were conducted by linear regression in which the outcomes were serum levels of total cholesterol, LDL, HDL, triglyceride and the total cholesterol/HDL ratio. Sex, BMI (in quartiles) and age were adjusted for in the models. Firstly, exposure groups based only on water districts (control area, recently and non-recent/uncertain exposure) were used as categorical variables. Then, within each exposure group, measured serum levels of PFOS, PFHxS, PFOA and a molar-weight adjusted sum of the 3 PFAS were used as categorical percentile variables (ventiles in the lower range up to the 20th percentile and deciles in the higher range) and continuous ln-transformed (natural log-transformed) variables separately. The purpose of the analysis of three exposure groups separately was to investigate is there a dose-response relation and whether the relation was similar in the three exposure groups.

Ln-transformed continuous variables, i.e. serum levels of PFAS, were used since from previous studies, a curvilinear association with less steep slope at high PFAS levels was expected [5]. We used ln-transformed values for the outcomes as it improved the linear model fitting. The improvement was particularly notable for triglyceride and the total cholesterol/HDL ratio. The interaction terms of PFAS and sex were tested for the possible varied associations between PFAS and lipids in different sex groups, which showed no clear evidence. In addition, in the lower range (i.e. restriction of each PFAS compound to the lower 40% of the distribution), where it is reasonable to assume a linear association, we did linear regressions for untransformed total cholesterol and LDL on untransformed PFAS variables.

In order to visualize the non-linear relationship between serum PFAS levels and serum lipids, generalized additive models [18] were used to produce smooth splines. In the generalized additive models, ln-transformed lipid levels were estimated as smooth functions of ln-transformed PFAS adjusted for BMI (in quartiles), sex, and age. The smooth functions of PFAS used thin-plate regression splines as its basis function as this tends to give the minimum mean square error [18]. The procedure generalized additive model (gam) in the ‘mgcv’ package in R was used.

In addition, in the two Ronneby-exposed groups (i.e. recently exposed group and non-recent/uncertain exposure group), the odds of high (> 6.2 mmol/L) serum cholesterol was examined using logistic regression on ln-transformed PFAS.

## Results

Table 1 shows descriptive statistics of measured serum PFAS, serum lipids and other characteristics in participants aged 20 to 60 years in each exposure group. Data for adults including those older than 60 years are shown in Supplementary Table S2. The distributions of the serum PFAS levels were skewed, especially in the two Ronneby exposed groups where the exposure was dominated by PFOS (median 157 ng/ml) and PFHxS (median 136 ng/ml). The median PFOA level was 8.6 ng/ml. The median levels of PFAS (i.e. PFOS, PFHxS and PFOA) in the exposed groups were 5 to 100-fold higher than in the control group. The serum PFAS levels in the control group were comparable to the general population in Sweden. The serum PFAS levels were markedly higher in the recently exposed group than in the group with non-recent/uncertain exposure, albeit with a large variation between individuals. The correlations between the serum PFAS levels are shown in Supplementary Table S3. The medians of total cholesterol, LDL, triglyceride and total cholesterol/HDL ratio were higher among participants from Ronneby than in the control group, while HDL levels were similar (Table 1). There was no difference in serum lipids between the recently exposed and non-recent/uncertain exposure groups. Median age, gender proportion and BMI were comparable across the different exposure groups.

**Table 1:** Descriptive statistics on the study group for adults 20 to 60 years

	Control group	Exposed groups		
		Total exposed group	Non-recent/uncertain exposure	Recently exposed
*N*	130	1815	655	1160
Median age	42	42	43	42
Proportion of males	43%	43%	44%	42%
Median BMI, kg/m^2^	25.1	25.9	25.7	26.0
PFOS	4.8 (2.0, 12)	160 (12, 590)	45 (5.6, 270)	240 (48, 660)
PFHxS	0.98 (0.38, 4.8)	140 (5.8, 560)	40 (2.1, 250)	210 (35, 630)
PFOA	1.6 (0.69, 3.5)	8.6 (1.3, 31)	3.5 (0.96, 14)	13 (2.7, 34)
sum PFAS^*a*^	0.017 (0.0076, 0.040)	0.68 (0.040, 2.6)	0.20 (0.021, 1.2)	1.0 (0.19, 2.9)
Total cholesterol	4.9 (3.7, 6.9)	5.2 (3.6, 8.2)	5.3 (3.7, 8.4)	5.2 (3.6, 8)
LDL	2.8 (1.9, 4.5)	3.1 (1.8, 5.3)	3.2 (1.9, 5.4)	3.1 (1.8, 5.3)
HDL	1.5 (0.86, 2.3)	1.5 (0.86, 2.5)	1.5 (0.88, 2.6)	1.4 (0.84, 2.5)
Triglycerides	1.1 (0.45, 3.6)	1.3 (0.60, 3.4)	1.3 (0.60, 3.4)	1.3 (0.50, 3.4)
Ratio^*b*^	3.2 (2.1, 6.5)	3.6 (2.2, 6.3)	3.5 (2.2, 6.3)	3.6 (2.2, 6.7)

Note: The units for PFOS, PFHxS and PFOA are ng/ml, the unit for sum PFAS is nmol/ml, The units for total cholesterol, LDL and HDL are mmol/L. The numbers presented for PFAS and blood lipids are medians and 5th, 95th percentiles in parentheses

^*a*^Sum PFAS: A molar-weight adjusted sum of PFOS, PFHxS and PFOA

^*b*^Ratio: the ratio of total cholesterol to HDL

The results from the linear model, adjusted for age, sex, and BMI, with exposure groups as a categorical variable indicated that both total cholesterol and LDL were clearly higher in the total Ronneby exposed group than in the control group (total cholesterol: 7% higher, *p* < 0.01, and LDL 9% higher, *p* < 0.01). The adjusted marginal means of lipid concentrations are shown in Table 2. There was little evidence of any difference between exposure groups for HDL, and the total cholesterol/HDL ratio and triglyceride. None of the serum lipids differed between the two Ronneby-exposed groups.

**Table 2:** Adjusted means of lipids in exposure groups among adults 20 to 60 years

	Control Group	Total exposed group	Exposed groups Not-recent/uncertain exposure	Recently exposed	*p*-values^*a*^
Total cholesterol, (95%CI)	4.93 (4.75, 5.13)	5.27 (5.21, 5.32)	5.28 (5.19, 5.38)	5.26 (5.19, 5.33)	< 0.01
LDL, (95% CI)	2.86 (2.72, 3.02)	3.12 (3.07, 3.16)	3.12 (3.05, 3.19)	3.12 (3.06, 3.17)	< 0.01
HDL, (95%CI)	1.39 (1.32, 1.46)	1.44 (1.42, 1.46)	1.45 (1.42, 1.48)	1.43 (1.40, 1.45)	0.20
Triglyceride, (95%CI)	1.24 (1.14, 1.35)	1.33 (1.30, 1.36)	1.35 (1.30, 1.41)	1.31 (1.28, 1.35)	0.14
Ratio^*b*^ (95% CI)(95% CI)	3.56 (3.38, 3.74)	3.67 (3.62, 3.72)	3.64 (3.56, 3.73)	3.68 (3.62, 3.74)	0.23

Note: The units for total cholesterol, LDL and HDL are mmol/L. The means were adjusted for age, sex and BMI in quartiles

^*a*^*p*-values are for the comparisons of adjusted means between the controls and the total exposed group

^*b*^Ratio: the ratio of total cholesterol to HDL

Linear regression showed positive associations with low *p*-values between serum levels of PFAS and serum levels of total cholesterol, most clearly in the recently exposed, and the slopes (per ln-unit of PFAS) were slightly higher in the control group (Table 3). The results for LDL were similar with slightly stronger positive associations. For HDL, similar positive slopes to LDL were evident, but the association in the much smaller unexposed group showed high *p*-values. Associations between triglyceride and PFAS, and between the total cholesterol/HDL ratio and PFAS were generally weak and close to null.

**Table 3:** Regression results of ln-transformed lipids versus ln-transformed PFAS among adults 20 to 60 years

		All (*n* = 1945)		Control (*n* = 130)		Total exposed (*n* = 1815)		Recently exposed (*n* = 1160)	
		Coefficent (SE)	*p*	Coefficent (SE)	*p*	Coefficent (SE)	*p*	Coefficent (SE)	*p*
Total cholesterol	PFOS	0.013 (0.0036)	< 0.01	0.058 (0.028)	0.04	0.0091 (0.0044)	0.04	0.035 (0.0089)	< 0.01
	PFHxS	0.011 (0.0029)	< 0.01	0.051 (0.021)	0.02	0.0072 (0.0039)	0.06	0.029 (0.0080)	< 0.01
	PFOA	0.016 (0.0050)	< 0.01	0.042 (0.033)	0.2	0.011 (0.0055)	0.05	0.035 (0.0094)	< 0.01
	Sum PFAS^*a*^	0.013 (0.0034)	< 0.01	0.075 (0.031)	0.02	0.0084 (0.0043)	0.05	0.032 (0.0086)	< 0.01
LDL	PFOS	0.017 (0.0049)	< 0.01	0.084 (0.041)	0.04	0.012 (0.0060)	0.05	0.050 (0.012)	< 0.01
	PFHxS	0.014 (0.0040)	< 0.01	0.087 (0.031)	0.01	0.0093 (0.0053)	0.08	0.043 (0.011)	< 0.01
	PFOA	0.020 (0.0068)	< 0.01	0.073 (0.048)	0.13	0.013 (0.0075)	0.09	0.047 (0.013)	< 0.01
	Sum PFAS	0.016 (0.0047)	< 0.01	0.12 (0.045)	0.01	0.011 (0.0058)	0.07	0.047 (0.012)	< 0.01
HDL	PFOS	0.013 (0.0046)	< 0.01	0.052 (0.034)	0.13	0.014 (0.0057)	0.01	0.045 (0.012)	< 0.01
	PFHxS	0.0097 (0.0038)	0.01	0.013 (0.026)	0.63	0.011 (0.0051)	0.03	0.036 (0.011)	< 0.01
	PFOA	0.019 (0.0065)	< 0.01	0.045 (0.040)	0.25	0.017 (0.0072)	0.02	0.048 (0.013)	< 0.01
	Sum PFAS	0.012 (0.0045)	0.01	0.040 (0.038)	0.29	0.013 (0.0056)	0.02	0.041 (0.012)	< 0.01
Triglyceride	PFOS	−0.0075 (0.0080)	0.35	−0.047 (0.080)	0.56	−0.021 (0.0098)	0.03	−0.039 (0.020)	0.05
	PFHxS	−0.0029 (0.0066)	0.65	−0.011 (0.061)	0.86	−0.015 (0.0086)	0.07	−0.032 (0.018)	0.08
	PFOA	− 0.011 (0.011)	0.32	− 0.058 (0.092)	0.53	−0.019 (0.012)	0.12	−0.018 (0.021)	0.39
	Sum PFAS	−0.0060 (0.0077)	0.44	−0.036 (0.088)	0.68	−0.018 (0.0095)	0.05	−0.035 (0.019)	0.07
total cholesterol/HDL ratio	PFOS	−0.00010 (0.0047)	0.98	0.0062 (0.044)	0.89	−0.0051 (0.0057)	0.37	−0.0099 (0.012)	0.40
	PFHxS	0.0011 (0.0038)	0.78	0.038 (0.033)	0.25	−0.0039 (0.0050)	0.44	−0.0072 (0.011)	0.50
	PFOA	−0.0030 (0.0065)	0.65	−0.0031 (0.050)	0.95	−0.0066 (0.0071)	0.35	−0.013 (0.012)	0.29
	Sum PFAS	0.00030 (0.0045)	0.95	0.035 (0.048)	0.47	−0.0046 (0.0055)	0.41	−0.0086 (0.011)	0.45

Note: All estimates are from regression models with ln-transformed lipids as response, ln-transformed of PFAS as the variables of interest and with additional adjusted for age, sex and BMI (in quartiles)

^*a*^Sum PFAS: Molar-weight adjusted sum of PFOS, PFHxS and PFOA

**Fig. 1:**
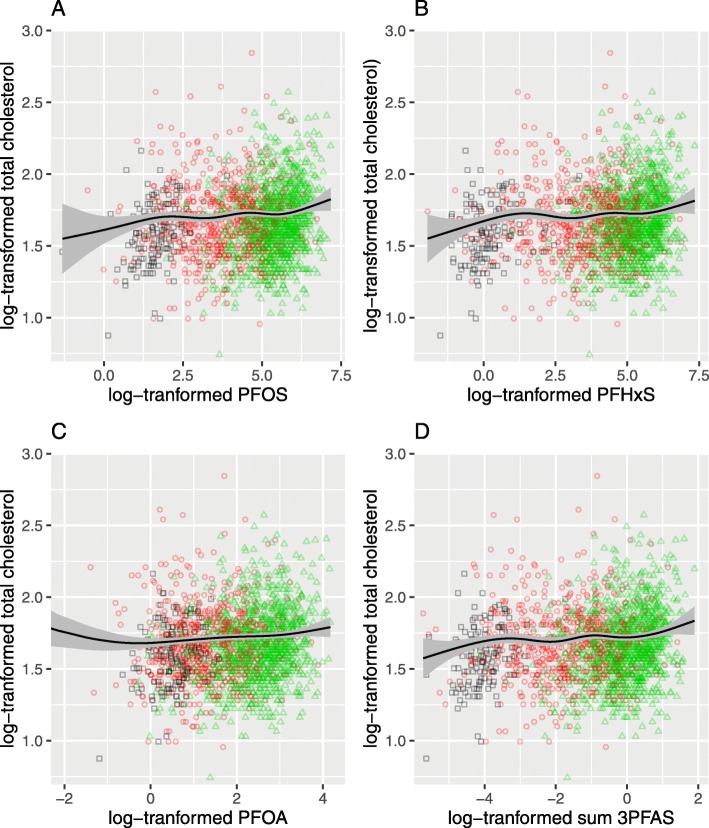
Smooth spline of ln-transformed total cholesterol with 95% confidence bands, in Ronneby, Sweden. *Legends*: **a** ln-transformed PFOS **b** ln-transformed PFHxS **c** ln-transformed PFOA and **d** ln-transformed a molar-weight adjusted sum of PFOS, PFHxS and PFOA. The smooth spline function was adjusted by age, sex and BMI in quartiles. The points on the graph are the original data with black squares are the ones from the control group, the red circles are the ones from the non-recent or uncertain group and the green triangles are the ones from the recently exposed group

**Fig. 2:**
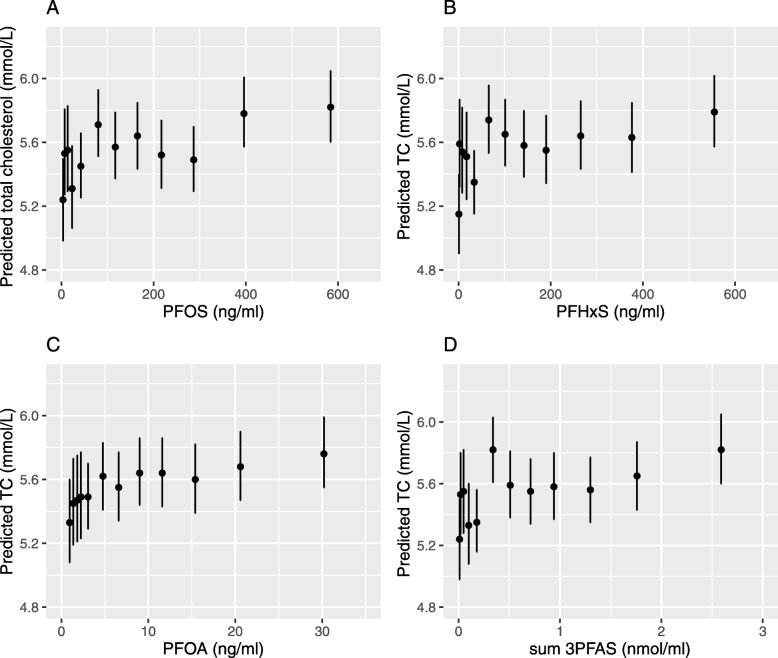
Adjusted means for total cholesterol in PFAS ventile (up to the 20th percentile) and deciles. *Legends*: **a** PFOS **b** PFHxS **c** PFOA and **d** sum 3 PFAS, which is a molar-weight adjusted sum of PFOS, PFHxS and PFOA, in Ronneby, Sweden. The means were adjusted with age, sex and BMI in quartiles

As shown by the splines in Fig. 1 the underlying linear slopes (on the ln-scale) identified in Table 3 were evident and in the predicted total cholesterol by ventiles or deciles of PFAS in Fig. 2, on a linear scale there was a suggestion of a steeper slope in the lower range then flattening off with little change at higher serum PFAS levels.

Visual inspection of Fig. 2, (in more detail in Tables S5 to S8), suggests a steeper slope up to about the 40th percentile, and the slope derived from the linear regression of cholesterol and LDL on serum levels of PFAS, restricted in each case to the lower 40% of the distribution, is shown in Table 4. The same regression model is also applied to the control population which has an even lower range of PFAS concentrations. PFOS, PFHxS and sum of PFAS all show clear associations with total cholesterol and LDL. In the control population, the slope coefficients are 10 to 20 times higher but most of the control group has serum levels below 12 ng/ml for PFOS and 5 ng/ml for PFHxS. Results for PFOA are imprecise though in the same direction. Results for the sum of PFAS are not directly comparable as units differ. These results confirm the pattern suggested by the plots that the slope per unit of PFAS serum concentration is steeper in the lower part of the exposure range, for PFOS and PFHxS below 100 ng/ ml and even steeper below about 10 ng/ml. Coefficients are broadly similar between individual PFAS, but their concentrations are highly correlated in this population.

**Table 4:** Regression results for total cholesterol and LDL restricted to below the PFAS 40th percentile separately

		All (*n* = 779)		Control (*n* = 130)	
		coefficent (SE)	*p*	coefficent (SE)	*P*
Total cholesterol	PFOS	0.0037 (0.0016)	0.02	0.038 (0.018)	0.04
	PFHxS	0.0043 (0.0018)	0.02	0.052 (0.026)	0.05
	PFOA	0.062 (0.035)	0.08	0.073 (0.095)	0.44
	Sum PFAS^*a*^	1.1 (0.36)	< 0.01	11 (4.8)	0.03
LDL	PFOS	0.0023 (0.0013)	0.07	0.041 (0.015)	0.01
	PFHxS	0.0032 (0.0015)	0.03	0.061 (0.021)	< 0.01
	PFOA	0.037 (0.028)	0.18	0.095 (0.079)	0.23
	Sum PFAS	0.73 (0.29)	0.01	12 (4.0)	< 0.01

Note: All the estimates are from linear models with total cholesterol or LDL as outcome, with un-transformed serum levels of PFAS as continuous variables, adjusted for sex, age and BMI (in quartiles). In the linear models checking the association with PFOS, the study group was restricted to the ones with serum PFOS level below the 40th percentile (99.7 ng/ml) ignoring other PFAS. For PFHxS, it was up to 81 ng/ml. For PFOA, it was up to 5.7 ng/ml. For sum PFAS, it was up to its 0.42 nmol/ml

^*a*^Sum PFAS: Molar-weight adjusted sum of PFOS, PFHxS and PFOA

The smooth splines in Fig. 1 are shown for other lipid fractions and for the subgroups of exposed and recently exposed in Supplementary Figs. S1-S20. The associations for HDL, triglyceride and the total cholesterol/HDL ratio (Supplementary Figs. S9–20) showed less apparent patterns than for total cholesterol or LDL (Supplementary Fig. S1-S8). This is also demonstrated when PFAS levels were stratified by ventiles or deciles (Supplementary Tables S7-16).

There were 13 and 22% of participants with high cholesterol (> 6.2 mmol/L) in the control and total exposed groups (*p* = 0.04). While there was little evidence of an association in the total exposed group, in the subgroup of recently exposed, logistic regression showed strong associations with the serum levels of PFOS, PFHxS, PFOA and the sum of the 3 PFAS (Table 5). The odds ratios for high cholesterol were about 1.3 for each unit increase in ln-PFAS, i.e. approximately a 2.7-fold increase. An analysis by quartiles showed increased odds ratios also in the total exposed group, most of which were, however, not statistically significant (data not shown).

**Table 5:** Results of logistic regression with high cholesterol in the total exposed and recently exposed groups

	Total exposed group		Recently exposed group	
	Odd Ratio (95% CI)	*p*	Odd Ratio (95% CI)	*p*
ln (PFOS)	1.06 (0.96, 1.17)	0.23	1.34 (1.09, 1.68)	0.01
ln (PFHxS)	1.05 (0.97, 1.15)	0.21	1.29 (1.07, 1.57)	0.01
ln (PFOA)	1.08 (0.96, 1.21)	0.23	1.35 (1.08, 1.70)	0.01
ln (Sum PFAS^*a*^)	1.06 (0.96, 1.16)	0.23	1.32 (1.08, 1.63)	0.01

Note: All estimates are from logistic regressions with high cholesterol (≥6.2 mmol/L) as outcome, and ln-transformed serum PFAS as continuous variables, adjusted for sex, age and BMI (in quartiles) in the total exposed group and recently exposed group. There are 1815 participants in the total exposed group with 397 of them having high cholesterol, and 1160 participants in the recently exposed group with 245 of them having high cholesterol

^*a*^Sum PFAS: Molar-weight adjusted sum of PFOS, PFHxS and PFOA

## Discussion

In the current study, participants were recruited from a community, in Sweden, polluted by aqueous film-forming foam with mixed PFAS exposure, predominantly PFOS and PFHxS. The serum levels of PFOS and PFHxS showed a wide span from background levels to the highest levels hitherto reported in peer reviewed publications with associated lipid findings in a local community. We found positive associations between PFAS exposure and serum lipids, especially total cholesterol and LDL. Total cholesterol and LDL levels were higher in the contaminated area than in the control area. Furthermore, there were clear positive associations between serum levels of PFAS (PFOS, PFHxS, PFOA, and the molar-weight adjusted sum of the 3 PFAS) and serum levels of total cholesterol, and LDL. These associations were observed especially in the recently exposed group, and for some associations they were significant also in the small control group. There was also an association between serum levels of PFAS and odds of high cholesterol (> 6.2 mmol/L).

Our findings regarding PFOS and PFOA are consistent with prior studies in the general population [5, 9–12, 19, 20], as reviewed by EFSA [7]. Few studies have, however, been performed on associations between PFHxS and serum lipids. A study of 2700 Canadian adults [8] found a positive association between serum PFHxS and total cholesterol, and LDL, while Nelson [9] observed inverse associations in 860 individuals from the US NHANES survey, and a study of 891 pregnant women in Norway found no association [10]. The levels of PFHxS were low in all these studies; medians about 2 ng/mL in the Canadian and US studies, and < 1 ng/mL in the Norwegian study. Therefore, given small study size, they had limited power to demonstrate if elevated PFHxS is associated with increased serum lipids. In our study the median level of PFHxS was 120 ng/ml and with a wide range.

The reason why the slope of the association between PFOS/PFHxS and cholesterol was higher in the lower range is unknown. This has been observed also by Steenland et al. [5] for PFOA and PFOS, and by Eriksen et al. [19] for PFOS, with a flattening off of the dose-response relation at serum PFAS levels above 50 ng/mL. Steenland et al. [5] found an increase of total cholesterol of about 4% when comparing individuals in the lowest strata of PFOS/PFOA with individuals having PFOS/PFOA levels of about 20 ng/mL. These slopes are similar to the slopes found in the controls of the present study. The fact that most of the increase in cholesterol occurs in the “normal range” for general populations has important implications for risk assessment since PFAS exposure is then relevant also in the general population without occupational exposure or other unusual exposure sources. The associations between serum levels of PFAS and serum levels of total cholesterol, and LDL in the control group were not always statistically significant which may be due to the small sample size.

Triglyceride levels were similar in the control and the exposed areas. Steenland et al. [5] found a positive association between serum PFOA and triglycerides, but almost all other studies found no such associations for PFOS or PFOA [7].

The comparison of serum lipid levels between the exposed and control areas was based only on water districts, not serum PFAS levels. Therefore, it is not affected by potential confounding related to intestinal reabsorption of PFAS and bile acids [15, 16]. However, the participants from the two areas may differ in other aspects than water quality. A relatively large fraction of people living in the contaminated area in Ronneby participated in the survey of the present study, while the participation rates in blood sampling were lower in the uncontaminated area in Ronneby and in the control area. Although the recruiting procedure in the control area was open to everyone, it is possible that people who were more interested in environmental issues, or in checking their health status were more prone to participate. About 30% of participants in the reference group were recruited from staff and students at a college, and the socio-economic status of the reference group may therefore be higher than that of the exposed area. Although we cannot exclude some ecological confounding in the comparison of cholesterol levels between water districts, the important advantage of this comparison is that it excludes any confounding by correlated intestinal reabsorption of PFAS and bile acids, since it does not rely on serum levels of PFAS. Consistent findings on increased cholesterol when quantifying exposure in terms of serum levels and water districts supports causality between exposure to PFAS and increased serum cholesterol.

In the present study, the analyses were adjusted for age, sex and BMI, which are the most important potential confounders. We have no information on diet, but confounding from dietary factors has been considered unlikely [7]. The cross-sectional design makes causal inference uncertain, and a longitudinal follow-up is desirable. In addition to previous cross-sectional studies, there is also a longitudinal study showing a significant positive association between the change of serum levels of PFOS and PFOA and change of serum cholesterol over a four-year follow-up period [12]. This and other longitudinal studies [13, 14] lends further support for causality. An argument presented against causality between PFAS and increasing cholesterol in humans is based on the observation that PFAS act as PPARα agonists in rodents leading to a PPARα-mediated reduction of serum lipids. However, the PPARα-mediated reduction of serum lipids occurs at very high exposure to PFAS, and the human PPARα seems less active than the rodent PPARα [7]. Furthermore, several other pathways seem to be relevant for the association between PFAS exposure and serum cholesterol in humans, as indicated for example by associations between serum PFOA levels and expression of genes involved in human cholesterol transport and metabolism [21].

We observed a 7–9% increase of total cholesterol and LDL in the PFAS contaminated area compared to the control area. A similar increase in cholesterol was noted over the low range of serum PFOS, PFHxS and PFOA, similar to the findings of Steenland et al. [5]. This is a clinically relevant increase in cholesterol. An increase of total cholesterol by 5%, even in the normal range, increases the risk of ischemic heart disease by at least 5% [22]. In addition, the odds of high cholesterol increased for all PFAS compounds measured. For example, in the Framingham study, high cholesterol (> 6.2 mmol/L) was associated with a relative risk of coronary heart disease of about 1.8 [23]. It should be noted, however, that we did not find any association between PFAS levels and the total cholesterol/HDL ratio. This ratio is considered a somewhat better indicator of cardiovascular risk that total and LDL cholesterol [22]. Few previous studies examined associations between PFAS and the ratio of total cholesterol to HDL. In the large C8 adult study, dominated by exposure to PFOA, there was a positive association with the total cholesterol/HDL ratio both for PFOS and PFOA [5]. A Canadian study in a general population [8] found a positive association with PFHxS and PFOS but not with PFOA, while a study on Inuit in Canada [24] found a negative association with PFOS. Two other studies [10, 25] reported positive associations between PFOS and total cholesterol, and positive associations with HDL cholesterol, without results on their ratio. Thus, the literature regarding the association between PFAS and the total cholesterol/HDL ratio is not consistent.

We performed separate analyses for PFOS, PFHxS, PFOA, and a molar-weight adjusted sum of the 3 PFAS. All of these PFAS compounds showed similar direction associations with serum lipids. However, we acknowledge that the high correlations mean we cannot disentangle these PFAS, especially PFOS and PFHxS (Spearman correlation: 0.75 in reference group and 0.9 in contaminated group).

Our study has limitations, mainly because of the lack of information on the use of cholesterol-lowering medication, dietary habits, and socioeconomic status, all of which affect serum lipids and are potential confounders if they happened to be associated with PFAS levels. Treatment with lipid-lowering medications will reduce the number of individuals with abnormal serum cholesterol. Our study also has a number of strengths. It includes a relatively large sample of study participants, a large range of serum PFAS levels, and a comparison between individuals in a PFAS-contaminated area and a reference area based only on residency addresses. It is also the first study with high exposure to PFHxS.

## Conclusions

In conclusion, the present study provides further evidence of a causal association between PFAS (PFOS, PFHxS and PFOA) and serum lipids.

## Supplementary information

**Additional file 1: ** Supplementary Material 1 **Table S1.** PFAS levels (ng/L) measured in outgoing drinking water from the two waterworks in Ronneby, Sweden on Dec 10, 2013. **Table S2.** Descriptive statistics on the study group for all adults age 20 years or more. **Table S3.** Spearman correlations of PFOS, PFHxS and PFOA in the control and the total exposed group. **Table S4.** Adjusted (for age, sex, BMI in quartiles) means of serum serum lipids in exposure groups among adults age 20 years or more. **Table S5-S8.** Serum lipids by ventiles (up to 20^th^ percentile) and deciles of PFOS, PFHxS, PFOA and sums of PFAS with 95% confidence interval in all adults 20 to 60 years. **Table S9-S12.** Serum lipids by ventiles (up to 20^th^ percentile) and deciles of PFOS, PFHxS, PFOA and sums of PFAS with 95% confidence interval in adults 20 to 60 years in total exposed areas. **Table S13-S16.** Serum lipids by deciles of PFOS, PFHxS, PFOA and sums of PFAS with 95% confidence interval in adults 20 to 60 years in recently exposed areas. **Figure S1-S4.** Smooth spline of total cholesterol on PFOS, PFHxS, PFOA, and the sum of PFAS. **Figure S5-S8.** Smooth spline of LDL on PFOS, PFHxS, PFOA, and the sum of PFAS. **Figure S9-S12.** Smooth spline of HDL on PFOS, PFHxS, PFOA, and the sum of PFAS. **Figure S13-S16.** Smooth spline of triglycerides on PFOS, PFHxS, PFOA, and the sum of PFASs. **Figure S17-S20.** Smooth spline of total cholesterol/HDL ratio on PFOS, PFHxS, PFOA and the sum of PFAS. Supplementary Material 2. Chemical analysis of PFAS.

## Data Availability

The data that support the findings of this study are available from the corresponding author upon reasonable request.

## Abbreviations

AFFF: Aqueous film-forming foam
BD: Becton Dickinson
BMI: Body mass index
HBM4EU: The Human Biomonitoring for Europe project
HDL: High-density lipoprotein
LC-MS/MS: Liquid chromatography coupled to tandem mass spectrometry
LDL: Low-density lipoprotein
PFAS: Perfluoroalkyl substances
PFHxS: Perfluorohexane sulfonic acid
PFOA: Perfluorooctanoic acid
PFOS: Perfluorooctane sulfonic acid
