# Supplementary material for: Associations between perfluoroalkyl substances and serum lipids in a Swedish adult population with contaminated drinking water
Source: Environ Health. 2020 Mar 14;19:33. doi: 10.1186/s12940-020-00588-9 (PMC7071576; doi:10.1186/s12940-020-00588-9)
Supplement: Supplementary file 1 — Additional file 1: Supplementary Material 1 Table S1. PFAS levels (ng/L) measured in outgoing drinking water from the two waterworks in Ronneby, Sweden on Dec 10, 2013. Table S2. Descriptive statistics on the study group for all adults age 20 years or more. Table S3. Spearman correlations of PFOS, PFHxS and PFOA in the control and the total exposed group. Table S4. Adjusted (for age, sex, BMI in quartiles) means of serum serum lipids in exposure groups among adults age 20 years or more. Table S5-S8. Serum lipids by ventiles (up to 20th percentile) and deciles of PFOS, PFHxS, PFOA and sums of PFAS with 95% confidence interval in all adults 20 to 60 years. Table S9-S12. Serum lipids by ventiles (up to 20th percentile) and deciles of PFOS, PFHxS, PFOA and sums of PFAS with 95% confidence interval in adults 20 to 60 years in total exposed areas. Table S13-S16. Serum lipids by deciles of PFOS, PFHxS, PFOA and sums of PFAS with 95% confidence interval in adults 20 to 60 years in recently exposed areas. Figure S1-S4. Smooth spline of total cholesterol on PFOS, PFHxS, PFOA, and the sum of PFAS. Figure S5-S8. Smooth spline of LDL on PFOS, PFHxS, PFOA, and the sum of PFAS. Figure S9-S12. Smooth spline of HDL on PFOS, PFHxS, PFOA, and the sum of PFAS. Figure S13-S16. Smooth spline of triglycerides on PFOS, PFHxS, PFOA, and the sum of PFASs. Figure S17-S20. Smooth spline of total cholesterol/HDL ratio on PFOS, PFHxS, PFOA and the sum of PFAS. Supplementary Material 2. Chemical analysis of PFAS. [file 12940_2020_588_MOESM1_ESM.docx]

**Supplementary Material**

**Associations between perfluoroalkyl substances and serum lipids in a Swedish adult population with contaminated drinking water**

Ying Li, Lars Barregard, Yiyi Xu, Kristin Scott, Daniela Pineda, Christian H. Lindh, Kristina Jakobsson, Tony Fletcher

**Table of Contents**

**Supplementary Material 1: Tables and Figures**

Table S1. PFAS levels (ng/L) measured in outgoing drinking water from the two waterworks in Ronneby, Sweden on Dec 10, 2013.

Table S2. Descriptive statistics on the study group for all adults age 20 years or more.

Table S3. Spearman correlations of PFOS, PFHxS and PFOA in the control and the total exposed group.

Table S4. Adjusted (for age, sex, BMI in quartiles) means of serum serum lipids in exposure groups among adults age 20 years or more.

Table S5-S8. Serum lipids by ventiles (up to 20^th^ percentile) and deciles of PFOS, PFHxS, PFOA and sums of PFAS with 95% confidence interval in all adults 20 to 60 years

Table S9-S12. Serum lipids by ventiles (up to 20^th^ percentile) and deciles of PFOS, PFHxS, PFOA and sums of PFAS with 95% confidence interval in adults 20 to 60 years in total exposed areas.

Table S13-S16 Serum lipids by deciles of PFOS, PFHxS, PFOA and sums of PFAS with 95% confidence interval in adults 20 to 60 years in recently exposed areas.

Figure S1-S4. Smooth spline of total cholesterol on PFOS, PFHxS, PFOA, and the sum of PFAS.

Figure S5-S8: Smooth spline of LDL on PFOS, PFHxS, PFOA, and the sum of PFAS.

Figure S9-S12: Smooth spline of HDL on PFOS, PFHxS, PFOA, and the sum of PFAS.

Figure S13-S16: Smooth spline of triglycerides on PFOS, PFHxS, PFOA, and the sum of PFASs.

Figure S17-S20: Smooth spline of total cholesterol/HDL ratio on PFOS, PFHxS, PFOA and the sum of PFAS.

**Supplementary Material 2: Chemical analysis of PFAS.**

References

***Supplementary material 1: Tables and Figures***

Table S1. PFAS levels (ng/L) measured in outgoing drinking water from the two waterworks in Ronneby, Sweden on Dec 10, 2013

|  | Brantafors | Kärragården |
| --- | --- | --- |
| Perfluoropentanoic acid, PFPeA | 38 | 10 |
| Perfluorohexanoic acid, PFHxA | 320 | 3.6 |
| Perfluoroheptanoic acid, PFHpA | 32 | 1.4 |
| Perfluorooctanoic acid, PFOA | 100 | 1.0 |
| Perfluorononanoic acid, PFNA | <1 | <1 |
| Perfluorodecanoic acid, PFDA | <1 | <1 |
| Perfluoroundecanoic acid, PFUnA | <10 | <10 |
| Perfluorododecanoic acid, PFDoA | <10 | <10 |
| Perfluorobutane sulfonic acid, PFBS | 130 | <2.6 |
| Perfluorohexane sulfonic acid, PFHxS | 1700 | 4.6 |
| Perfluoroheptane sulfonic acid, PFHpS | 60 | <1 |
| Perfluorooctane sulfonic acid, PFOS | 8000 | 27 |

Table S2. Descriptive statistics on the study group for all adults age 20 years or more

|  | Control group | Total exposed group | Non-recent/uncertain exposure | Recently exposed |
| --- | --- | --- | --- | --- |
| N | 130 | 2471 | 828 | 1643 |
| Median age | 42 | 48 | 48 | 49 |
| Proportion of male | 0.43 | 0.43 | 0.44 | 0.43 |
| Median BMI, kg/m^2^ | 25.1 | 26.0 | 25.9 | 26.0 |
| Median PFOS, ng/ml (5%, 95%) | 4.76(1.95, 11.7) | 191 (12.9, 754) | 49.7(6.02, 308) | 290(54.8, 832) |
| Median PFHxS, ng/ml (5%, 95%) | 0.98(0.38, 4.78) | 166 (6.95, 754) | 45.0(2.13, 294) | 270(42.6, 817) |
| Median PFOA, ng/ml (5%, 95%) | 1.62(0.69, 3.48) | 10.4 (1.44, 38.8) | 3.71(0.98, 18.0) | 15.5(3.29, 42.5) |
| Median sum PFAS^#^ (5%, 95%) | 0.02(0.01, 0.04) | 0.82 (0.05, 3.49) | 0.22(0.02, 1.44) | 1.33(0.23, 3.78) |
| Median cholesterol, mmol/L (5%, 95%) | 4.9(3.65, 6.86) | 5.4 (3.6, 8.6) | 5.4(3.7, 8.8) | 5.4(3.6, 8.5) |
| Median LDL, mmol/L (5%, 95%) | 2.8(1.85, 4.5) | 3.2 (1.8, 5.6) | 3.25(1.9, 5.7) | 3.2(1.8, 5.6) |
| Median HDL, mmol/L (5%, 95%) | 1.5(0.86, 2.26) | 1.5 (0.86, 2.6) | 1.5(0.88, 2.6) | 1.5(0.85, 2.6) |
| Median triglyceride, mmol/L (5%, 95%) | 1.1(0.45, 3.61) | 1.4 (0.6, 3.6) | 1.4(0.6, 3.67) | 1.4(0.6, 3.5) |
| Median total cholesterol/HDL ratio, (5%, 95%) | 3.2(2.1, 6.53) | 3.6(2.16, 6.71) | 3.5(2.19, 6.75) | 3.6(2.16, 6.7) |

^#^Sum PFAS: Molar-adjusted sum 3PFAS.

Table S3. Spearman correlations of PFOS, PFHxS and PFOA in control and total exposed group

|  | Control group | | Total exposed group | |  |
| --- | --- | --- | --- | --- | --- |
|  | PFOS | PFHxs | PFOS | PFHxs | |
| PFHxs | 0.7 |  | 0.9 |  | |
| PFOA | 0.6 | 0.6 | 0.9 | 0.9 | |

Table S4. Adjusted (for age, sex and BMI in quartiles) means of serum lipids in exposure groups among adults age 20 years or more.

|  | Control group | Total exposed group | Non-recent/uncertain exposure | Recently exposed |
| --- | --- | --- | --- | --- |
| Adj.mean Cholesterol (95%CI) | 5.07(4.86, 5.29) | 5.38(5.33, 5.43) | 5.48(5.39, 5.57) | 5.33(5.27, 5.4) |
| Adj. mean LDL(95% CI) | 2.93(2.76, 3.11) | 3.16(3.12, 3.2) | 3.22(3.14, 3.29) | 3.13(3.08, 3.18) |
| Adj.mean HDL(95%CI) | 1.41(1.34, 1.49) | 1.46(1.44, 1.48) | 1.49(1.46, 1.52) | 1.45(1.43, 1.47) |
| Adj. mean Triglyceride(95%CI) | 1.31(1.2, 1.43) | 1.39(1.37, 1.42) | 1.44(1.39, 1.49) | 1.37(1.34, 1.41) |
| Adj.mean cholesterol ratio (95% CI) | 3.59 (3.41, 3.79) | 3.68 (3.64, 3.73) | 3.68 (3.61, 3,76) | 3.68 (3.63, 3.74) |

Table S5. Serum lipids by ventiles (up to 20^th^ percentile) and deciles of PFOS with 95% confidence interval in all adults 20 to 60 years. The adjusted means were based on the predicted lipid levels for female, 45 years old and BMI between 25.8 and 28.96 kg/m^2^, using linear model with ln-transformed lipids as outcomes, categorical PFOS variable and adjusted with covariates

| PFOS | n | PFOS median (range) | Adjusted mean total cholesterol (95% CI) | Adjusted mean LDL (95% CI) | Adjusted mean HDL (95% CI) | Adjusted mean Triglycerides  (95% CI) | Adjusted mean total cholesterol/HDL ratio (95% CI) |
| --- | --- | --- | --- | --- | --- | --- | --- |
| 0-5% | 98 | 3.45 (0.27, 4.87) | 5.24 (4.98, 5.5) | 3.03 (2.84, 3.25) | 1.52 (1.43, 1.62) | 1.27 (1.13, 1.42) | 3.44 (3.23, 3.67) |
| 5-10% | 97 | 7.07 (4.87, 9.7) | 5.53 (5.27, 5.81) | 3.22 (3.01, 3.44) | 1.54 (1.45, 1.64) | 1.43 (1.28, 1.59) | 3.59 (3.37, 3.83) |
| 10-15% | 97 | 13.5 (9.7, 17.3) | 5.55 (5.29, 5.83) | 3.31 (3.1, 3.54) | 1.58 (1.48, 1.68) | 1.27 (1.14, 1.42) | 3.52 (3.31, 3.75) |
| 15-20% | 97 | 23.3 (17.3, 29.1) | 5.31 (5.06, 5.58) | 3.05 (2.85, 3.27) | 1.52 (1.43, 1.62) | 1.43 (1.28, 1.59) | 3.5 (3.28, 3.73) |
| 20-30% | 195 | 42.2 (29.1, 58.9) | 5.45 (5.25, 5.66) | 3.2 (3.04, 3.37) | 1.53 (1.46, 1.61) | 1.36 (1.25, 1.48) | 3.56 (3.38, 3.74) |
| 30-40% | 194 | 79.8 (58.9, 99.7) | 5.71 (5.51, 5.93) | 3.34 (3.17, 3.51) | 1.62 (1.54, 1.7) | 1.41 (1.3, 1.53) | 3.54 (3.37, 3.71) |
| 40-50% | 194 | 117 (99.7, 144) | 5.57 (5.37, 5.79) | 3.3 (3.13, 3.47) | 1.58 (1.5, 1.65) | 1.39 (1.28, 1.51) | 3.54 (3.37, 3.72) |
| 50-60% | 195 | 165 (144, 188) | 5.64 (5.43, 5.85) | 3.28 (3.11, 3.45) | 1.62 (1.54, 1.7) | 1.33 (1.22, 1.45) | 3.48 (3.32, 3.66) |
| 60-70% | 194 | 217 (188, 252) | 5.52 (5.31, 5.74) | 3.31 (3.14, 3.49) | 1.58 (1.5, 1.66) | 1.26 (1.16, 1.38) | 3.5 (3.33, 3.68) |
| 70-80% | 195 | 287 (252, 336) | 5.49 (5.29, 5.7) | 3.25 (3.08, 3.42) | 1.63 (1.55, 1.71) | 1.21 (1.11, 1.31) | 3.37 (3.21, 3.54) |
| 80-90% | 194 | 396 (336, 468) | 5.78 (5.57, 6.01) | 3.43 (3.26, 3.62) | 1.58 (1.51, 1.67) | 1.36 (1.25, 1.48) | 3.65 (3.47, 3.84) |
| 90-100% | 195 | 584 (468, 1301) | 5.82 (5.6, 6.05) | 3.43 (3.26, 3.62) | 1.65 (1.57, 1.74) | 1.34 (1.23, 1.46) | 3.52 (3.35, 3.71) |

Table S6. Serum lipids by ventiles (up to 20^th^ percentile) and deciles of PFHxS with 95% confidence interval in all adults 20 to 60 years. The adjusted means were based on the predicted lipid levels for female, 45 years old and BMI between 25.8 and 28.96 kg/m^2^, using linear model with ln-transformed lipids as outcomes, categorical PFOS variable and adjusted with covariates

| PFHxS | n | PFHxS median (range) | Adjusted mean total cholesterol (95% CI) | Adjusted mean LDL (95% CI) | Adjusted mean HDL (95% CI) | Adjusted mean Triglycerides  (95% CI) | Adjusted mean total cholesterol/HDL ratio (95% CI) | |
| --- | --- | --- | --- | --- | --- | --- | --- | --- |
| 0-5% | 98 | 0.75 (0.14, 1.24) | 5.15 (4.9, 5.4) | 2.92 (2.73, 3.12) | 1.55 (1.46, 1.65) | 1.26 (1.13, 1.41) | 3.32 (3.11, 3.54) |  |
| 5-10% | 97 | 2.19 (1.24, 4.39) | 5.59 (5.32, 5.87) | 3.34 (3.12, 3.57) | 1.51 (1.42, 1.61) | 1.35 (1.21, 1.51) | 3.69 (3.46, 3.93) |  |
| 10-15% | 97 | 8 (4.39, 12.17) | 5.54 (5.28, 5.82) | 3.3 (3.09, 3.53) | 1.55 (1.45, 1.65) | 1.32 (1.18, 1.47) | 3.58 (3.36, 3.81) |  |
| 15-20% | 97 | 17.6 (12.2, 22.2) | 5.51 (5.24, 5.79) | 3.14 (2.93, 3.36) | 1.62 (1.52, 1.72) | 1.39 (1.25, 1.56) | 3.41 (3.19, 3.63) |  |
| 20-30% | 195 | 34 (22.2, 47.6) | 5.35 (5.15, 5.55) | 3.07 (2.92, 3.23) | 1.52 (1.45, 1.6) | 1.39 (1.28, 1.51) | 3.51 (3.34, 3.69) |  |
| 30-40% | 194 | 65.6 (47.6, 81.3) | 5.74 (5.53, 5.96) | 3.44 (3.27, 3.62) | 1.57 (1.5, 1.65) | 1.44 (1.33, 1.57) | 3.65 (3.48, 3.83) |  |
| 40-50% | 194 | 101 (81.3, 121) | 5.65 (5.45, 5.87) | 3.29 (3.13, 3.46) | 1.65 (1.57, 1.73) | 1.35 (1.24, 1.47) | 3.43 (3.27, 3.6) |  |
| 50-60% | 195 | 142 (121, 165) | 5.58 (5.38, 5.8) | 3.3 (3.14, 3.48) | 1.58 (1.5, 1.66) | 1.32 (1.22, 1.44) | 3.54 (3.37, 3.71) |  |
| 60-70% | 194 | 190 (165, 221) | 5.55 (5.34, 5.77) | 3.33 (3.16, 3.51) | 1.59 (1.51, 1.67) | 1.25 (1.14, 1.36) | 3.49 (3.32, 3.67) |  |
| 70-80% | 195 | 265 (221, 318) | 5.64 (5.43, 5.86) | 3.35 (3.18, 3.53) | 1.64 (1.56, 1.72) | 1.25 (1.14, 1.36) | 3.44 (3.27, 3.61) |  |
| 80-90% | 194 | 376 (318, 447) | 5.63 (5.41, 5.85) | 3.31 (3.14, 3.49) | 1.6 (1.52, 1.69) | 1.27 (1.17, 1.39) | 3.51 (3.34, 3.69) |  |
| 90-100% | 195 | 555 (447, 1550) | 5.79 (5.57, 6.02) | 3.41 (3.23, 3.59) | 1.63 (1.55, 1.71) | 1.39 (1.28, 1.52) | 3.56 (3.38, 3.74) |  |

Table S7. Serum lipids by ventiles (up to 20^th^ percentile) and deciles of PFOA with 95% confidence interval in all adults 20 to 60 years. The adjusted means were based on the predicted lipid levels for female, 45 years old and BMI between 25.8 and 28.96 kg/m^2^, using linear model with ln-transformed lipids as outcomes, categorical PFOS variable and adjusted with covariates

| PFOA | n | PFOA median (range) | Adjusted mean total cholesterol (95% CI) | Adjusted mean LDL (95% CI) | Adjusted mean HDL (95% CI) | Adjusted mean Triglycerides  (95% CI) | Adjusted mean total cholesterol/HDL ratio (95% CI) |
| --- | --- | --- | --- | --- | --- | --- | --- |
| 0-5% | 98 | 0.95 (0.04, 1.2) | 5.33 (5.08, 5.6) | 3.08 (2.88, 3.29) | 1.51 (1.41, 1.61) | 1.42 (1.27, 1.58) | 3.54 (3.32, 3.77) |
| 5-10% | 97 | 1.37 (1.2, 1.6) | 5.45 (5.19, 5.73) | 3.25 (3.04, 3.48) | 1.52 (1.43, 1.62) | 1.25 (1.12, 1.4) | 3.58 (3.36, 3.82) |
| 10-15% | 97 | 1.83 (1.6, 2.05) | 5.47 (5.21, 5.75) | 3.16 (2.95, 3.38) | 1.58 (1.49, 1.69) | 1.34 (1.2, 1.5) | 3.45 (3.24, 3.68) |
| 15-20% | 97 | 2.25 (2.05, 2.5) | 5.49 (5.23, 5.77) | 3.16 (2.95, 3.38) | 1.55 (1.46, 1.65) | 1.45 (1.3, 1.62) | 3.54 (3.32, 3.77) |
| 20-30% | 195 | 3.06 (2.5, 3.86) | 5.49 (5.29, 5.7) | 3.23 (3.06, 3.4) | 1.57 (1.5, 1.65) | 1.33 (1.22, 1.45) | 3.49 (3.32, 3.66) |
| 30-40% | 194 | 4.78 (3.86, 5.72) | 5.62 (5.41, 5.83) | 3.31 (3.14, 3.48) | 1.59 (1.52, 1.67) | 1.35 (1.24, 1.47) | 3.53 (3.36, 3.7) |
| 40-50% | 194 | 6.59 (5.72, 7.81) | 5.55 (5.34, 5.77) | 3.25 (3.09, 3.43) | 1.58 (1.5, 1.66) | 1.35 (1.24, 1.47) | 3.52 (3.35, 3.7) |
| 50-60% | 195 | 9 (7.81, 10.2) | 5.64 (5.44, 5.86) | 3.35 (3.18, 3.52) | 1.56 (1.49, 1.64) | 1.4 (1.29, 1.53) | 3.61 (3.44, 3.79) |
| 60-70% | 194 | 11.6 (10.2, 13.5) | 5.64 (5.43, 5.86) | 3.35 (3.18, 3.53) | 1.61 (1.53, 1.69) | 1.27 (1.17, 1.39) | 3.51 (3.34, 3.69) |
| 70-80% | 195 | 15.4 (13.5, 17.8) | 5.6 (5.39, 5.82) | 3.31 (3.14, 3.49) | 1.66 (1.58, 1.74) | 1.22 (1.12, 1.33) | 3.38 (3.21, 3.55) |
| 80-90% | 194 | 20.6 (17.8, 23.8) | 5.68 (5.47, 5.9) | 3.37 (3.2, 3.55) | 1.6 (1.52, 1.68) | 1.33 (1.23, 1.45) | 3.56 (3.39, 3.74) |
| 90-100% | 195 | 30.2 (23.8, 64.7) | 5.76 (5.55, 5.99) | 3.36 (3.19, 3.55) | 1.65 (1.57, 1.74) | 1.35 (1.24, 1.47) | 3.49 (3.32, 3.67) |

Table S8. Serum lipids by ventiles (up to 20^th^ percentile) and deciles of the sum of PFAS with 95% confidence interval in all adults 20 to 60 years. The adjusted means were based on the predicted lipid levels for female, 45 years old and BMI between 25.8 and 28.96 kg/m^2^, using linear model with ln-transformed lipids as outcomes, categorical PFOS variable and adjusted with covariates

| Sum PFAS*^a^* | n | PFAS median (range) | Adjusted mean total cholesterol (95% CI) | Adjusted mean LDL (95% CI) | Adjusted mean HDL (95% CI) | Adjusted mean Triglycerides  (95% CI) | Adjusted mean total cholesterol/HDL ratio (95% CI) |
| --- | --- | --- | --- | --- | --- | --- | --- |
| 0-5% | 98 | 0.01 (0, 0.02) | 5.24 (4.98,5.5) | 3 (2.8,3.21) | 1.55 (1.46, 1.66) | 1.29 (1.15, 1.44) | 3.37 (3.16, 3.6) |
| 5-10% | 97 | 0.02 (0.02, 0.04) | 5.53 (5.27, 5.8) | 3.26 (3.05, 3.48) | 1.51 (1.41, 1.61) | 1.39 (1.24, 1.55) | 3.67 (3.44, 3.91) |
| 10-15% | 97 | 0.05 (0.04, 0.07) | 5.55 (5.28, 5.82) | 3.34 (3.13, 3.57) | 1.56 (1.47, 1.67) | 1.27 (1.14, 1.42) | 3.55 (3.33, 3.78) |
| 15-20% | 97 | 0.1 (0.07, 0.12) | 5.33 (5.08, 5.6) | 3 (2.8, 3.21) | 1.55 (1.45, 1.65) | 1.43 (1.28, 1.6) | 3.44 (3.23, 3.67) |
| 20-30% | 195 | 0.18 (0.12, 0.25) | 5.35 (5.16, 5.56) | 3.11 (2.96, 3.28) | 1.51 (1.44, 1.59) | 1.37 (1.26, 1.49) | 3.55 (3.38, 3.73) |
| 30-40% | 194 | 0.34 (0.25, 0.42) | 5.82 (5.61, 6.03) | 3.44 (3.28, 3.62) | 1.63 (1.56, 1.71) | 1.42 (1.3, 1.54) | 3.56 (3.4, 3.74) |
| 40-50% | 194 | 0.51 (0.42, 0.61) | 5.59 (5.38, 5.81) | 3.28 (3.11, 3.45) | 1.6 (1.53, 1.68) | 1.36 (1.25, 1.49) | 3.49 (3.32, 3.66) |
| 50-60% | 195 | 0.71 (0.61, 0.82) | 5.55 (5.34, 5.76) | 3.23 (3.07, 3.4) | 1.58 (1.51, 1.66) | 1.33 (1.22, 1.45) | 3.5 (3.33, 3.68) |
| 60-70% | 194 | 0.94 (0.82, 1.09) | 5.58 (5.37, 5.8) | 3.36 (3.19, 3.54) | 1.6 (1.52, 1.68) | 1.27 (1.16, 1.38) | 3.48 (3.31, 3.66) |
| 70-80% | 195 | 1.3 (1.09, 1.5) | 5.56 (5.35, 5.77) | 3.26 (3.09, 3.43) | 1.66 (1.58, 1.75) | 1.21 (1.11, 1.32) | 3.34 (3.18, 3.51) |
| 80-90% | 194 | 1.76 (1.5, 2.1) | 5.65 (5.43, 5.87) | 3.35 (3.18, 3.53) | 1.58 (1.51, 1.66) | 1.29 (1.19, 1.41) | 3.57 (3.39, 3.75) |
| 90-100% | 195 | 2.59 (2.1, 6.63) | 5.82 (5.6, 6.05) | 3.43 (3.26, 3.62) | 1.61 (1.53, 1.69) | 1.41 (1.29,1.54) | 3.62 (3.44, 3.81) |

*^a^*Sum PFAS: Molar-adjusted sum 3PFAS.

Table S9. Serum lipids by ventiles (up to 20^th^ percentile) and deciles of PFOS with 95% confidence interval in total exposed areas. The adjusted means were based on the predicted lipid levels for female, 45 years old and BMI between 25.8 and 28.96 kg/m^2^, using linear model with ln-transformed lipids as outcomes, categorical PFOS variable and adjusted with covariates.

| PFOS | n | | PFOS median (range) | | Adjusted mean total cholesterol (95% CI) | | Adjusted mean LDL (95% CI) | | Adjusted mean HDL (95% CI) | | Adjusted mean Triglycerides  (95% CI) | | Adjusted mean total cholesterol/HDL ratio (95% CI) | |
| --- | --- | --- | --- | --- | --- | --- | --- | --- | --- | --- | --- | --- | --- | --- |
| PFOS | n | median(range), ng/ml | | Adjusted mean total cholesterol, mmol/L, (95%) | | Adjusted mean LDL, mmol/L, (95%) | | Adjusted mean HDL, mmol/L, (95%) | | Adjusted mean Triglyceride, mmol/L, (95%) | | Adjusted mean total cholesterol/HDL  (95%) | |  |
| 0-5% | 91 | 6.8 (0.6, 11) | | 5.74 (5.45, 6.04) | | 3.4 (3.17, 3.64) | | 1.56 (1.46, 1.67) | | 1.47 (1.31, 1.64) | | 3.67 (3.43, 3.92) | |  |
| 5-10% | 91 | 14.8 (11, 19.5) | | 5.53 (5.26, 5.81) | | 3.3 (3.08, 3.54) | | 1.55 (1.45, 1.66) | | 1.32 (1.18, 1.47) | | 3.56 (3.34, 3.81) | |  |
| 10-15% | 91 | 25.3 (19.5, 31.2) | | 5.26 (4.99, 5.54) | | 2.99 (2.79, 3.21) | | 1.51 (1.41, 1.61) | | 1.43 (1.28, 1.6) | | 3.49 (3.27, 3.74) | |  |
| 15-20% | 90 | 37 (31.2, 43.7) | | 5.52 (5.24, 5.82) | | 3.23 (3, 3.47) | | 1.57 (1.46, 1.68) | | 1.4 (1.25, 1.57) | | 3.53 (3.3, 3.78) | |  |
| 20-30% | 182 | 58.9 (43.7, 78) | | 5.45 (5.24, 5.67) | | 3.17 (3, 3.34) | | 1.58 (1.5, 1.66) | | 1.33 (1.22, 1.45) | | 3.46 (3.29, 3.64) | |  |
| 30-40% | 181 | 96.3 (78, 114.3) | | 5.8 (5.58, 6.03) | | 3.43 (3.26, 3.62) | | 1.61 (1.53, 1.7) | | 1.47 (1.34, 1.6) | | 3.59 (3.42, 3.78) | |  |
| 40-50% | 181 | 138.7 (114.3, 157.4) | | 5.6 (5.38, 5.83) | | 3.27 (3.1, 3.46) | | 1.6 (1.52, 1.69) | | 1.37 (1.26, 1.5) | | 3.5 (3.32, 3.68) | |  |
| 50-60% | 182 | 178.4 (157.4, 203.1) | | 5.55 (5.33, 5.77) | | 3.26 (3.09, 3.44) | | 1.58 (1.5, 1.66) | | 1.28 (1.18, 1.4) | | 3.52 (3.34, 3.7) | |  |
| 60-70% | 181 | 229.9 (203.1, 268.1) | | 5.54 (5.32, 5.77) | | 3.34 (3.16, 3.53) | | 1.6 (1.52, 1.69) | | 1.26 (1.15, 1.38) | | 3.46 (3.28, 3.65) | |  |
| 70-80% | 182 | 308.5 (268.1, 352.2) | | 5.55 (5.34, 5.77) | | 3.26 (3.09, 3.44) | | 1.63 (1.55, 1.72) | | 1.25 (1.14, 1.36) | | 3.4 (3.23, 3.58) | |  |
| 80-90% | 181 | 409.5 (352.2, 479.7) | | 5.75 (5.53, 5.98) | | 3.41 (3.23, 3.6) | | 1.59 (1.51, 1.68) | | 1.35 (1.23, 1.47) | | 3.61 (3.43, 3.8) | |  |
| 90-100% | 181 | 593.1 (479.7, 1301) | | 5.84 (5.6, 6.08) | | 3.43 (3.25, 3.63) | | 1.65 (1.56, 1.73) | | 1.37 (1.25, 1.49) | | 3.55 (3.37, 3.74) | |  |

Table S10. Serum lipids by ventiles (up to 20^th^ percentile) and deciles of PFHxS with 95% confidence interval in total exposed areas. The adjusted means were based on the predicted lipid levels for female, 45 years old and BMI between 25.8 and 28.96 kg/m^2^, using linear model with ln-transformed lipids as outcomes, categorical PFHxS variable and adjusted with covariates.

| PFHxS | n | median(range), ng/ml | Adjusted mean total cholesterol, mmol/L, (95%) | Adjusted mean LDL, mmol/L, (95%) | Adjusted mean HDL, mmol/L, (95%) | Adjusted mean Triglyceride, mmol/L, (95%) | Adjusted mean total cholesterol/HDL  (95%) |
| --- | --- | --- | --- | --- | --- | --- | --- |
| 0-5% | 91 | 2.8 (0.1, 5.8) | 5.58 (5.3, 5.88) | 3.31 (3.09, 3.55) | 1.52 (1.42, 1.63) | 1.41 (1.26, 1.58) | 3.67 (3.43, 3.92) |
| 5-10% | 91 | 9.5 (5.8, 13.9) | 5.64 (5.36, 5.93) | 3.34 (3.12, 3.58) | 1.57 (1.47, 1.68) | 1.38 (1.24, 1.55) | 3.6 (3.37, 3.84) |
| 10-15% | 91 | 18.7 (13.9, 23.5) | 5.33 (5.06, 5.62) | 3.04 (2.83, 3.27) | 1.58 (1.48, 1.69) | 1.34 (1.19, 1.51) | 3.37 (3.15, 3.61) |
| 15-20% | 90 | 28.2 (23.5, 34.9) | 5.43 (5.16, 5.72) | 3.17 (2.96, 3.4) | 1.48 (1.38, 1.58) | 1.47 (1.31, 1.65) | 3.67 (3.44, 3.93) |
| 20-30% | 182 | 47.6 (34.9, 64.3) | 5.49 (5.28, 5.71) | 3.14 (2.98, 3.31) | 1.6 (1.52, 1.69) | 1.36 (1.25, 1.48) | 3.42 (3.25, 3.6) |
| 30-40% | 181 | 79.3 (64.3, 95.5) | 5.73 (5.51, 5.95) | 3.45 (3.28, 3.64) | 1.58 (1.5, 1.66) | 1.39 (1.27, 1.51) | 3.62 (3.45, 3.81) |
| 40-50% | 181 | 113.7 (95.5, 136) | 5.57 (5.35, 5.8) | 3.21 (3.04, 3.39) | 1.64 (1.56, 1.73) | 1.34 (1.23, 1.46) | 3.4 (3.23, 3.58) |
| 50-60% | 182 | 155.8 (136, 178.7) | 5.59 (5.37, 5.81) | 3.3 (3.13, 3.49) | 1.59 (1.51, 1.67) | 1.32 (1.21, 1.45) | 3.52 (3.34, 3.7) |
| 60-70% | 181 | 204.5 (178.7, 233.5) | 5.69 (5.47, 5.92) | 3.44 (3.26, 3.63) | 1.61 (1.53, 1.69) | 1.27 (1.16, 1.38) | 3.54 (3.36, 3.72) |
| 70-80% | 182 | 283.1 (233.5, 333.3) | 5.56 (5.34, 5.79) | 3.27 (3.1, 3.45) | 1.61 (1.52, 1.69) | 1.29 (1.18, 1.41) | 3.46 (3.29, 3.65) |
| 80-90% | 181 | 389.6 (333.3, 457.2) | 5.65 (5.43, 5.88) | 3.34 (3.16, 3.53) | 1.6 (1.51, 1.68) | 1.28 (1.17, 1.4) | 3.54 (3.36, 3.73) |
| 90-100% | 181 | 560.6 (457.2, 1550.5) | 5.8 (5.57, 6.04) | 3.4 (3.22, 3.59) | 1.64 (1.56, 1.73) | 1.38 (1.26, 1.51) | 3.53 (3.35, 3.72) |

Table S11. Serum lipids by ventiles (up to 20^th^ percentile) and deciles of PFOA with 95% confidence interval in total exposed areas. The adjusted means were based on the predicted lipid levels for female, 45 years old and BMI between 25.8 and 28.96 kg/m^2^, using linear model with ln-transformed lipids as outcomes, categorical PFOA variable and adjusted with covariates.

| PFOA | n | median(range), ng/ml | Adjusted mean total cholesterol, mmol/L, (95%) | Adjusted mean LDL, mmol/L, (95%) | Adjusted mean HDL, mmol/L, (95%) | Adjusted mean Triglyceride, mmol/L, (95%) | Adjusted mean total cholesterol/HDL  (95%) |
| --- | --- | --- | --- | --- | --- | --- | --- |
| 0-5% | 91 | 1 (0, 1.3) | 5.51 (5.23, 5.8) | 3.24 (3.03, 3.48) | 1.54 (1.44, 1.64) | 1.39 (1.24, 1.55) | 3.59 (3.36, 3.83) |
| 5-10% | 91 | 1.6 (1.3, 2) | 5.47 (5.2, 5.75) | 3.21 (3, 3.44) | 1.51 (1.41, 1.61) | 1.37 (1.22, 1.53) | 3.63 (3.39, 3.87) |
| 10-15% | 91 | 2.2 (2, 2.4) | 5.59 (5.31, 5.89) | 3.21 (2.99, 3.44) | 1.59 (1.49, 1.7) | 1.48 (1.32, 1.66) | 3.52 (3.29, 3.76) |
| 15-20% | 90 | 2.8 (2.4, 3) | 5.64 (5.36, 5.94) | 3.37 (3.14, 3.62) | 1.59 (1.49, 1.7) | 1.35 (1.2, 1.51) | 3.55 (3.32, 3.79) |
| 20-30% | 182 | 3.8 (3, 4.7) | 5.4 (5.19, 5.62) | 3.17 (3, 3.34) | 1.56 (1.48, 1.64) | 1.27 (1.16, 1.39) | 3.46 (3.29, 3.64) |
| 30-40% | 181 | 5.6 (4.7, 6.4) | 5.71 (5.49, 5.94) | 3.35 (3.17, 3.53) | 1.62 (1.54, 1.71) | 1.41 (1.29, 1.54) | 3.52 (3.35, 3.71) |
| 40-50% | 181 | 7.5 (6.4, 8.6) | 5.55 (5.34, 5.78) | 3.26 (3.08, 3.44) | 1.57 (1.49, 1.65) | 1.4 (1.28, 1.53) | 3.53 (3.36, 3.72) |
| 50-60% | 182 | 9.7 (8.6, 10.8) | 5.61 (5.39, 5.84) | 3.35 (3.17, 3.53) | 1.55 (1.48, 1.64) | 1.34 (1.23, 1.46) | 3.61 (3.43, 3.8) |
| 60-70% | 181 | 12.5 (10.8, 14.3) | 5.66 (5.43, 5.89) | 3.34 (3.16, 3.53) | 1.64 (1.56, 1.73) | 1.28 (1.17, 1.4) | 3.45 (3.28, 3.64) |
| 70-80% | 182 | 16.1 (14.3, 18.6) | 5.61 (5.39, 5.84) | 3.31 (3.13, 3.5) | 1.67 (1.58, 1.76) | 1.22 (1.12, 1.34) | 3.36 (3.19, 3.54) |
| 80-90% | 181 | 21.1 (18.6, 24.6) | 5.7 (5.48, 5.93) | 3.38 (3.2, 3.56) | 1.61 (1.53, 1.69) | 1.35 (1.23, 1.47) | 3.55 (3.37, 3.74) |
| 90-100% | 181 | 31.1 (24.6, 64.7) | 5.77 (5.54, 6.01) | 3.37 (3.19, 3.56) | 1.64 (1.56, 1.73) | 1.37 (1.25, 1.5) | 3.52 (3.34, 3.71) |

Table S12. Serum lipids by ventiles (up to 20^th^ percentile) and deciles of the sum of PFAS with 95% confidence interval in total exposed areas. The adjusted means were based on the predicted lipid levels for female, 45 years old and BMI between 25.8 and 28.96 kg/m^2^, using linear model with ln-transformed lipids as outcomes, categorical total number PFAS variable and adjusted with covariates.

| Sum PFAS*^a^* | n | median(range) | Adjusted mean total cholesterol, mmol/L, (95%) | Adjusted mean LDL, mmol/L, (95%) | Adjusted mean HDL, mmol/L, (95%) | Adjusted mean Triglyceride, mmol/L, (95%) | Adjusted mean total cholesterol/HDL  (95%) |
| --- | --- | --- | --- | --- | --- | --- | --- |
| 0-5% | 91 | 0.02 (0.003, 0.04) | 5.65 (5.37, 5.95) | 3.32 (3.09, 3.56) | 1.55 (1.45, 1.66) | 1.47 (1.31, 1.64) | 3.64 (3.41, 3.89) |
| 5-10% | 91 | 0.1 (0, 0.1) | 5.57 (5.3, 5.86) | 3.35 (3.13, 3.6) | 1.53 (1.43, 1.64) | 1.32 (1.18, 1.47) | 3.64 (3.41, 3.89) |
| 10-15% | 91 | 0.1 (0.1, 0.1) | 5.34 (5.07, 5.62) | 3.02 (2.82, 3.25) | 1.57 (1.47, 1.68) | 1.43 (1.27, 1.6) | 3.41 (3.18, 3.64) |
| 15-20% | 90 | 0.2 (0.1, 0.2) | 5.41 (5.14, 5.7) | 3.19 (2.97, 3.43) | 1.51 (1.41, 1.61) | 1.35 (1.2, 1.52) | 3.59 (3.35, 3.84) |
| 20-30% | 182 | 0.2 (0.2, 0.3) | 5.51 (5.3, 5.73) | 3.17 (3.01, 3.34) | 1.58 (1.5, 1.66) | 1.39 (1.28, 1.52) | 3.48 (3.31, 3.66) |
| 30-40% | 181 | 0.4 (0.3, 0.5) | 5.78 (5.56, 6.01) | 3.47 (3.29, 3.66) | 1.62 (1.54, 1.7) | 1.4 (1.28, 1.53) | 3.58 (3.4, 3.76) |
| 40-50% | 181 | 0.6 (0.5, 0.7) | 5.6 (5.38, 5.82) | 3.24 (3.07, 3.42) | 1.61 (1.53, 1.7) | 1.38 (1.26, 1.5) | 3.48 (3.3, 3.66) |
| 50-60% | 182 | 0.8 (0.7, 0.9) | 5.53 (5.31, 5.75) | 3.25 (3.08, 3.43) | 1.59 (1.51, 1.67) | 1.27 (1.16, 1.39) | 3.48 (3.31, 3.66) |
| 60-70% | 181 | 1 (0.9, 1.2) | 5.6 (5.38, 5.83) | 3.38 (3.2, 3.57) | 1.59 (1.51, 1.67) | 1.29 (1.18, 1.41) | 3.52 (3.35, 3.71) |
| 70-80% | 182 | 1.4 (1.2, 1.6) | 5.59 (5.37, 5.82) | 3.29 (3.11, 3.47) | 1.66 (1.58, 1.75) | 1.21 (1.11, 1.32) | 3.37 (3.2, 3.54) |
| 80-90% | 181 | 1.8 (1.6, 2.2) | 5.69 (5.46, 5.92) | 3.35 (3.17, 3.54) | 1.57 (1.49, 1.66) | 1.34 (1.23, 1.47) | 3.62 (3.44, 3.82) |
| 90-100% | 181 | 2.6 (2.2, 6.6) | 5.81 (5.58, 6.05) | 3.41 (3.23, 3.61) | 1.64 (1.56, 1.73) | 1.39 (1.27, 1.53) | 3.54 (3.36, 3.74) |

*^a^*Sum PFAS: Molar-adjusted sum 3PFAS.

Table S13. Serum lipids by deciles of PFOS with 95% confidence interval in the recently exposed area. The adjusted means were based on the predicted lipid levels for female, 45 years old and BMI between 25.8 and 28.96 kg/m^2^, using linear model with ln-transformed lipids as outcomes, deciles of PFOS as categorical variable and adjusted with covariates.

| PFOS | n | median(range), ng/ml | Adjusted mean total cholesterol, mmol/L, (95%) | Adjusted mean LDL, mmol/L, (95%) | Adjusted mean HDL, mmol/L, (95%) | Adjusted mean Triglyceride, mmol/L, (95%) | Adjusted mean total cholesterol/HDL  (95%) |
| --- | --- | --- | --- | --- | --- | --- | --- |
| 0-10% | 116 | 47.5 (3.7, 73.4) | 5.14 (4.89, 5.4) | 2.89 (2.7, 3.09) | 1.47 (1.37, 1.57) | 1.41 (1.26, 1.58) | 3.51 (3.29, 3.74) |
| 10-20% | 116 | 100.4 (73.4, 117.9) | 5.64 (5.37, 5.91) | 3.32 (3.11, 3.55) | 1.55 (1.46, 1.66) | 1.47 (1.32, 1.64) | 3.63 (3.4, 3.87) |
| 20-30% | 116 | 143.8 (117.9, 158.6) | 5.58 (5.31, 5.86) | 3.31 (3.09, 3.54) | 1.57 (1.47, 1.67) | 1.34 (1.2, 1.5) | 3.56 (3.34, 3.8) |
| 30-40% | 116 | 175.5 (158.6, 194) | 5.41 (5.15, 5.68) | 3.15 (2.95, 3.37) | 1.54 (1.44, 1.65) | 1.29 (1.16, 1.44) | 3.51 (3.29, 3.74) |
| 40-50% | 116 | 215.3 (194, 236.8) | 5.51 (5.25, 5.79) | 3.35 (3.13, 3.59) | 1.56 (1.46, 1.67) | 1.27 (1.13, 1.42) | 3.53 (3.31, 3.77) |
| 50-60% | 116 | 262 (236.8, 286.3) | 5.42 (5.16, 5.69) | 3.27 (3.06, 3.5) | 1.61 (1.51, 1.72) | 1.13 (1.01, 1.26) | 3.36 (3.15, 3.58) |
| 60-70% | 116 | 319.4 (286.3, 351.1) | 5.51 (5.24, 5.78) | 3.18 (2.97, 3.4) | 1.65 (1.55, 1.77) | 1.26 (1.13, 1.41) | 3.33 (3.12, 3.55) |
| 70-80% | 116 | 384.9 (351.1, 428.4) | 5.72 (5.44, 6) | 3.34 (3.12, 3.58) | 1.61 (1.5, 1.72) | 1.38 (1.24, 1.55) | 3.56 (3.34, 3.8) |
| 80-90% | 116 | 479 (428.4, 552.1) | 5.63 (5.36, 5.92) | 3.38 (3.16, 3.62) | 1.59 (1.48, 1.7) | 1.27 (1.14, 1.42) | 3.55 (3.33, 3.79) |
| 90-100% | 115 | 660 (552.1, 1301) | 5.94 (5.66, 6.25) | 3.5 (3.27, 3.75) | 1.7 (1.59, 1.81) | 1.37 (1.23, 1.53) | 3.51 (3.28, 3.74) |

Table S14. Serum lipids by deciles of PFHxS with 95% confidence interval in the recently exposed area. The adjusted means were based on the predicted lipid levels for female, 45 years old and BMI between 25.8 and 28.96 kg/m^2^, using linear model with ln-transformed lipids as outcomes, deciles of PFHxS as categorical variable and adjusted with covariates.

| PFHxS | n | median(range), ng/ml | Adjusted mean total cholesterol, mmol/L, (95%) | Adjusted mean LDL, mmol/L, (95%) | Adjusted mean HDL, mmol/L, (95%) | Adjusted mean Triglyceride, mmol/L, (95%) | Adjusted mean total cholesterol/HDL  (95%) |
| --- | --- | --- | --- | --- | --- | --- | --- |
| 0-10% | 116 | 34.5 (2.6, 56.1) | 5.13 (4.88, 5.39) | 2.85 (2.66, 3.05) | 1.45 (1.36, 1.55) | 1.44 (1.29, 1.61) | 3.54 (3.32, 3.78) |
| 10-20% | 116 | 74.9 (56.1, 92.4) | 5.61 (5.35, 5.89) | 3.36 (3.15, 3.59) | 1.56 (1.46, 1.66) | 1.4 (1.25, 1.55) | 3.6 (3.38, 3.84) |
| 20-30% | 116 | 112.6 (92.4, 130.7) | 5.45 (5.19, 5.72) | 3.13 (2.92, 3.34) | 1.59 (1.49, 1.7) | 1.33 (1.19, 1.48) | 3.43 (3.22, 3.66) |
| 30-40% | 116 | 148.5 (130.7, 165.6) | 5.53 (5.27, 5.8) | 3.31 (3.1, 3.54) | 1.53 (1.44, 1.63) | 1.33 (1.19, 1.48) | 3.61 (3.39, 3.84) |
| 40-50% | 116 | 185 (165.6, 207.9) | 5.42 (5.15, 5.7) | 3.29 (3.06, 3.53) | 1.55 (1.44, 1.65) | 1.22 (1.09, 1.36) | 3.51 (3.28, 3.75) |
| 50-60% | 116 | 227.6 (207.9, 259.1) | 5.67 (5.41, 5.95) | 3.37 (3.15, 3.6) | 1.71 (1.6, 1.82) | 1.19 (1.07, 1.33) | 3.32 (3.11, 3.53) |
| 60-70% | 116 | 294.3 (259.1, 332.3) | 5.56 (5.29, 5.84) | 3.29 (3.07, 3.52) | 1.58 (1.48, 1.69) | 1.32 (1.18, 1.48) | 3.53 (3.3, 3.77) |
| 70-80% | 116 | 364.5 (332.3, 406.1) | 5.62 (5.35, 5.9) | 3.29 (3.08, 3.53) | 1.68 (1.58, 1.8) | 1.22 (1.09, 1.36) | 3.34 (3.13, 3.56) |
| 80-90% | 116 | 456.8 (406.1, 515.5) | 5.57 (5.3, 5.86) | 3.31 (3.09, 3.55) | 1.51 (1.41, 1.61) | 1.36 (1.22, 1.52) | 3.7 (3.46, 3.95) |
| 90-100% | 115 | 634.8 (515.5, 1550.5) | 5.86 (5.57, 6.16) | 3.43 (3.2, 3.68) | 1.67 (1.56, 1.79) | 1.41 (1.26, 1.58) | 3.51 (3.28, 3.75) |

Table S15. Serum lipids by deciles of PFOA with 95% confidence interval in the recently exposed area. The adjusted means were based on the predicted lipid levels for female, 45 years old and BMI between 25.8 and 28.96 kg/m^2^, using linear model with ln-transformed lipids as outcomes, deciles of PFOA as categorical variable and adjusted with covariates.

| PFOA | n | Median (Range), ng/ml | Adjusted mean total cholesterol, mmol/L, (95%) | Adjusted mean LDL, mmol/L, (95%) | Adjusted mean HDL, mmol/L, (95%) | Adjusted mean Triglyceride, mmol/L, (95%) | Adjusted mean total cholesterol/HDL  (95%) |
| --- | --- | --- | --- | --- | --- | --- | --- |
| 0-10% | 116 | 2.7 (0.7, 4.2) | 5.22 (4.97, 5.49) | 2.97 (2.78, 3.18) | 1.46 (1.37, 1.56) | 1.41 (1.26, 1.57) | 3.57 (3.35, 3.81) |
| 10-20% | 116 | 5.4 (4.2, 6.2) | 5.59 (5.32, 5.86) | 3.3 (3.08, 3.53) | 1.55 (1.45, 1.65) | 1.36 (1.22, 1.51) | 3.61 (3.38, 3.84) |
| 20-30% | 116 | 7.3 (6.2, 8.4) | 5.32 (5.06, 5.59) | 3.09 (2.88, 3.31) | 1.56 (1.46, 1.67) | 1.3 (1.17, 1.46) | 3.41 (3.2, 3.64) |
| 30-40% | 116 | 9.3 (8.4, 10.4) | 5.65 (5.38, 5.93) | 3.42 (3.2, 3.66) | 1.52 (1.43, 1.63) | 1.37 (1.23, 1.53) | 3.71 (3.48, 3.95) |
| 40-50% | 116 | 11.4 (10.4, 12.7) | 5.64 (5.37, 5.93) | 3.35 (3.13, 3.59) | 1.62 (1.52, 1.73) | 1.25 (1.12, 1.4) | 3.48 (3.26, 3.72) |
| 50-60% | 116 | 14 (12.7, 15.3) | 5.43 (5.17, 5.7) | 3.2 (2.99, 3.43) | 1.63 (1.53, 1.74) | 1.23 (1.1, 1.37) | 3.33 (3.12, 3.55) |
| 60-70% | 116 | 16.7 (15.3, 18.4) | 5.52 (5.25, 5.8) | 3.25 (3.03, 3.48) | 1.65 (1.55, 1.77) | 1.21 (1.08, 1.35) | 3.34 (3.13, 3.57) |
| 70-80% | 116 | 19.9 (18.4, 22) | 5.64 (5.37, 5.92) | 3.37 (3.15, 3.6) | 1.57 (1.47, 1.68) | 1.3 (1.17, 1.46) | 3.58 (3.36, 3.82) |
| 80-90% | 116 | 24.5 (22, 27.8) | 5.69 (5.42, 5.97) | 3.29 (3.07, 3.52) | 1.67 (1.56, 1.78) | 1.35 (1.21, 1.51) | 3.4 (3.19, 3.63) |
| 90-100% | 115 | 34 (27.8, 64.7) | 5.81 (5.53, 6.1) | 3.44 (3.21, 3.69) | 1.65 (1.54, 1.76) | 1.39 (1.24, 1.55) | 3.53 (3.31, 3.77) |

Table S16. Serum lipids by deciles of the sum of PFAS with 95% confidence interval in the recently expose areas. The adjusted means were based on the predicted lipid levels for female, 45 years old and BMI between 25.8 and 28.96 kg/m^2^, using linear model with ln-transformed lipids as outcomes, deciles of total number of PFAS as categorical variable and adjusted with covariates.

| Sum PFAS*^a^* | n | median(range) | Adjusted mean total cholesterol, mmol/L, (95%) | Adjusted mean LDL, mmol/L, (95%) | Adjusted mean HDL, mmol/L, (95%) | Adjusted mean Triglyceride, mmol/L, (95%) | Adjusted mean total cholesterol/HDL  (95%) |
| --- | --- | --- | --- | --- | --- | --- | --- |
| 0-10% | 116 | 0.2 (0, 0.3) | 5.15 (4.9, 5.41) | 2.9 (2.71, 3.1) | 1.49 (1.39, 1.59) | 1.39 (1.24, 1.55) | 3.47 (3.25, 3.7) |
| 10-20% | 116 | 0.4 (0.3, 0.5) | 5.64 (5.38, 5.92) | 3.34 (3.13, 3.57) | 1.54 (1.45, 1.65) | 1.45 (1.31, 1.62) | 3.66 (3.43, 3.89) |
| 20-30% | 116 | 0.6 (0.5, 0.7) | 5.52 (5.26, 5.8) | 3.21 (3, 3.44) | 1.58 (1.48, 1.69) | 1.35 (1.21, 1.51) | 3.5 (3.28, 3.74) |
| 30-40% | 116 | 0.7 (0.7, 0.8) | 5.42 (5.17, 5.69) | 3.21 (3, 3.43) | 1.51 (1.42, 1.61) | 1.29 (1.16, 1.44) | 3.59 (3.37, 3.82) |
| 40-50% | 116 | 0.9 (0.8, 1) | 5.58 (5.31, 5.87) | 3.37 (3.15, 3.61) | 1.6 (1.5, 1.71) | 1.24 (1.11, 1.39) | 3.49 (3.26, 3.72) |
| 50-60% | 116 | 1.2 (1, 1.3) | 5.42 (5.17, 5.69) | 3.25 (3.04, 3.48) | 1.61 (1.51, 1.72) | 1.17 (1.05, 1.31) | 3.37 (3.16, 3.59) |
| 60-70% | 116 | 1.4 (1.3, 1.6) | 5.6 (5.33, 5.88) | 3.27 (3.05, 3.5) | 1.71 (1.6, 1.82) | 1.23 (1.1, 1.37) | 3.28 (3.08, 3.5) |
| 70-80% | 116 | 1.7 (1.6, 1.9) | 5.79 (5.51, 6.08) | 3.41 (3.18, 3.65) | 1.6 (1.5, 1.71) | 1.41 (1.26, 1.58) | 3.62 (3.39, 3.87) |
| 80-90% | 116 | 2.1 (1.9, 2.4) | 5.49 (5.22, 5.77) | 3.25 (3.04, 3.49) | 1.54 (1.44, 1.64) | 1.31 (1.17, 1.46) | 3.58 (3.35, 3.82) |
| 90-100% | 115 | 2.9 (2.4, 6.6) | 5.95 (5.66, 6.26) | 3.5 (3.26, 3.75) | 1.72 (1.61, 1.84) | 1.38 (1.23, 1.54) | 3.47 (3.25, 3.71) |

*^a^*Sum PFAS: a molar-weight adjusted sum of PFOS, PFHxS and PFOA.


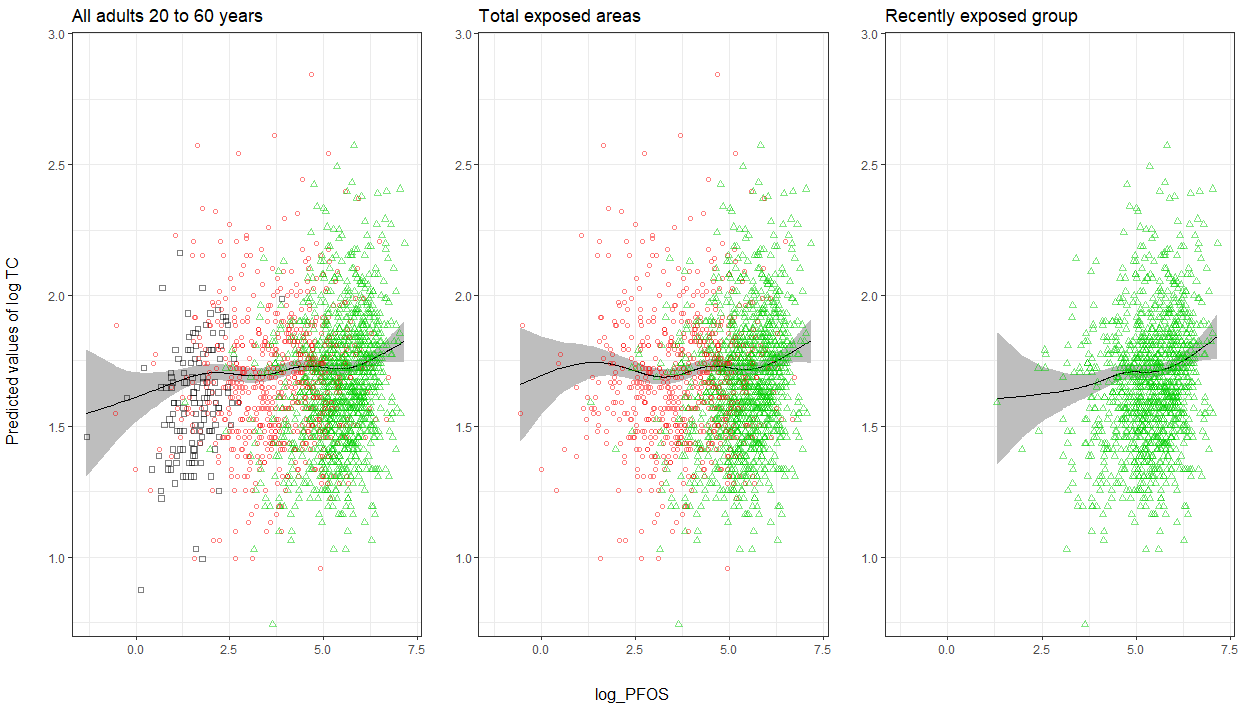


Figure S1: Smooth spline of ln-transformed total cholesterol with 95% confidence bands, in Ronneby, Sweden, on ln-transformed PFOS. The smooth spline function was adjusted by age, sex and BMI in quartiles. The points on the graph are the original data with black squares are the ones from the control group, the red circles are the ones from the non-recent or uncertain group and the green triangles are the ones from the recently exposed group.


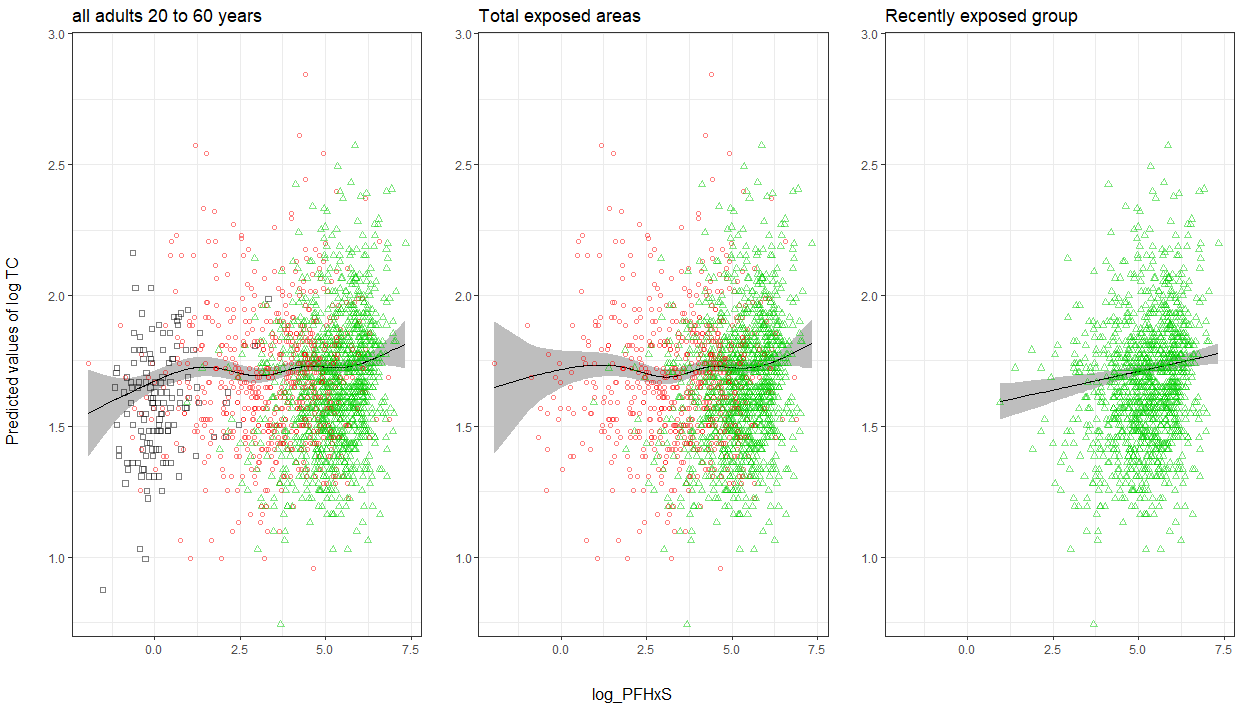


Figure S2: Smooth spline of ln-transformed total cholesterol with 95% confidence bands, in Ronneby, Sweden, on ln-transformed PFHxS. The smooth spline function was adjusted by age, sex and BMI in quartiles. The points on the graph are the original data with black squares are the ones from the control group, the red circles are the ones from the non-recent or uncertain group and the green triangles are the ones from the recently exposed group.


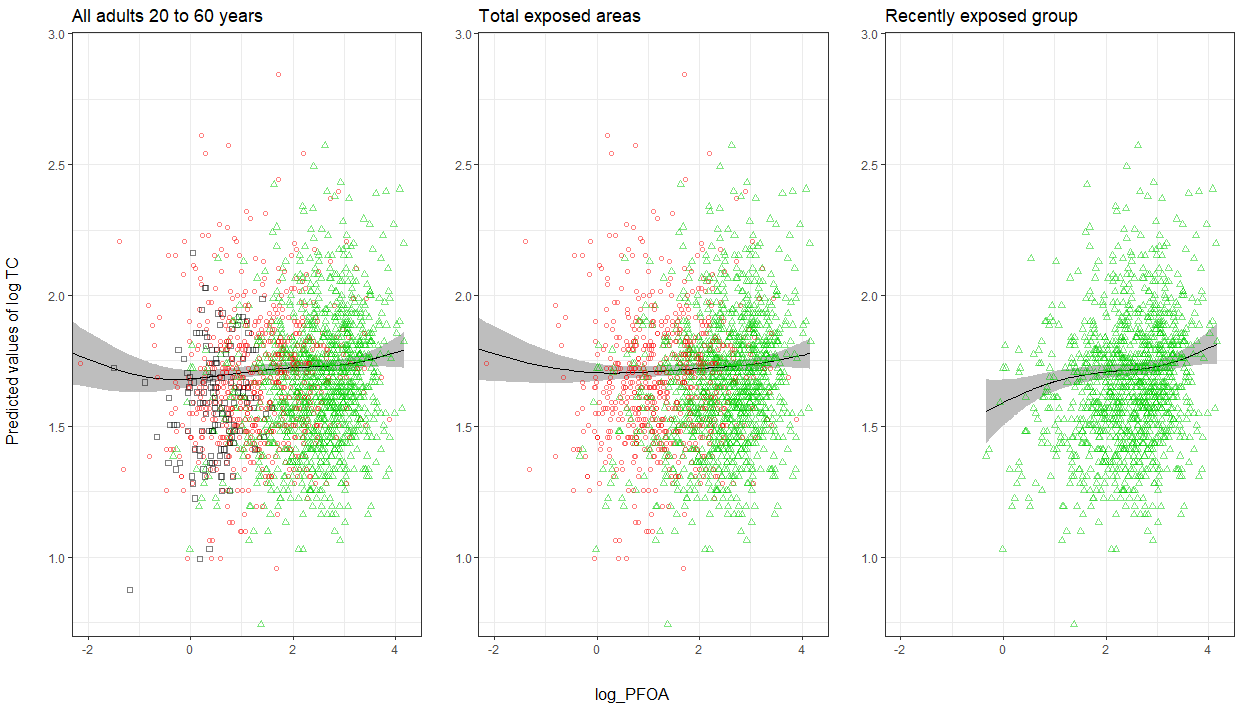


Figure S3. Smooth spline of ln-transformed total cholesterol with 95% confidence bands, in Ronneby, Sweden, on ln-transformed PFOA. The smooth spline function was adjusted by age, sex and BMI in quartiles. The points on the graph are the original data with black squares are the ones from the control group, the red circles are the ones from the non-recent or uncertain group and the green triangles are the ones from the recently exposed group.


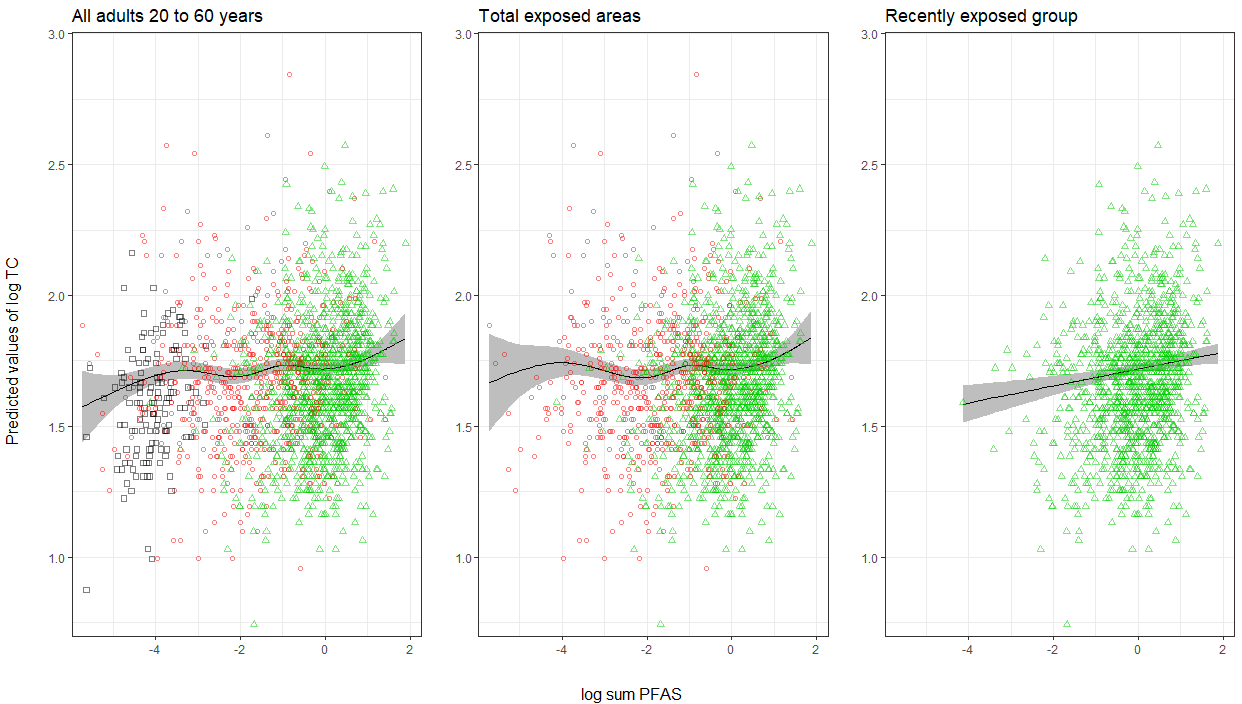


Figure S4. Smooth spline of ln-transformed total cholesterol with 95% confidence bands, in Ronneby, Sweden, on ln-transformed sum PFAS. The smooth spline function was adjusted by age, sex and BMI in quartiles. The points on the graph are the original data with black squares are the ones from the control group, the red circles are the ones from the non-recent or uncertain group and the green triangles are the ones from the recently exposed group.


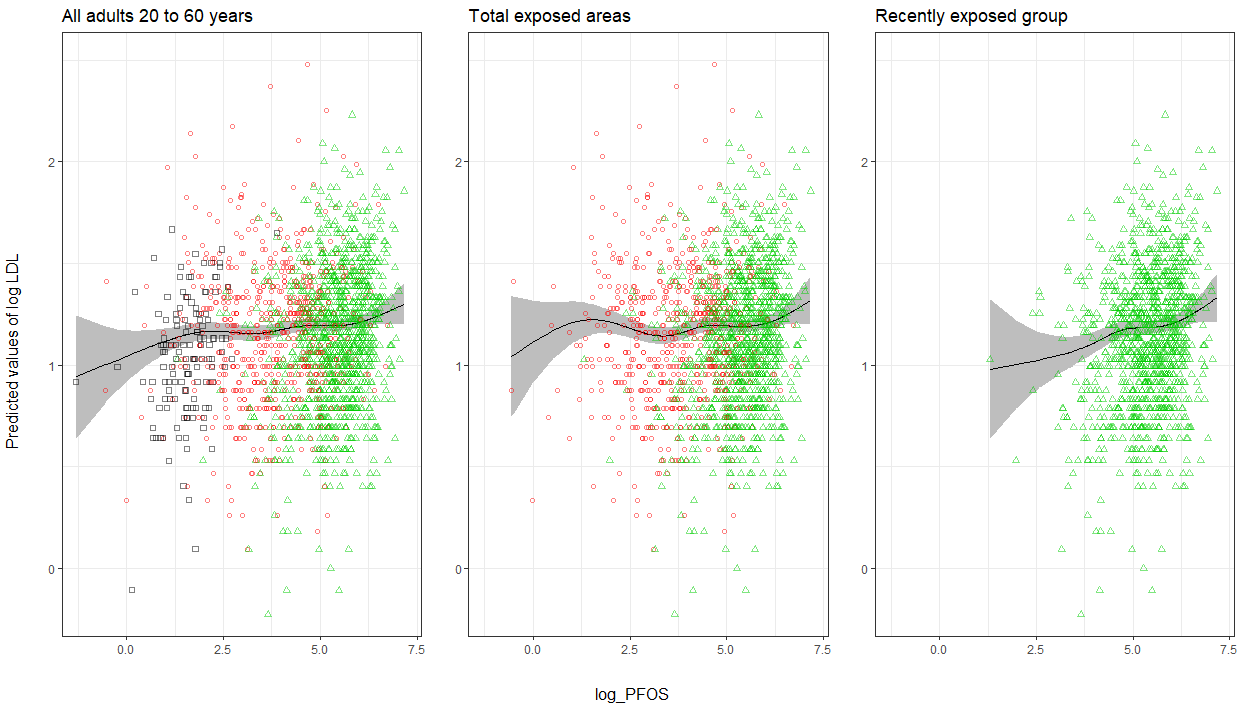


Figure S5. Smooth spline of ln-transformed LDL with 95% confidence bands, in Ronneby, Sweden, on ln-transformed PFOS. The smooth spline function was adjusted by age, sex and BMI in quartiles. The points on the graph are the original data with black squares are the ones from the control group, the red circles are the ones from the non-recent or uncertain group and the green triangles are the ones from the recently exposed group.


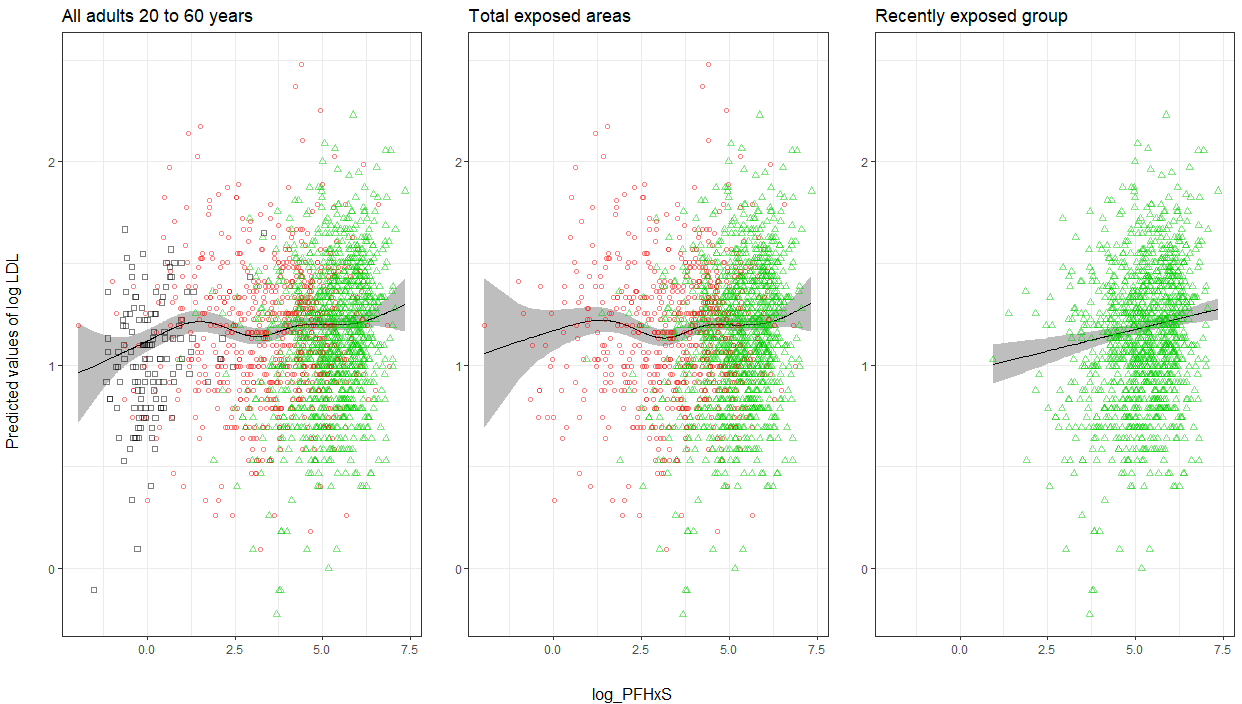


Figure S6 Smooth spline of ln-transformed LDL with 95% confidence bands, in Ronneby, Sweden, on ln-transformed PFHxS. The smooth spline function was adjusted by age, sex and BMI in quartiles. The points on the graph are the original data with black squares are the ones from the control group, the red circles are the ones from the non-recent or uncertain group and the green triangles are the ones from the recently exposed group.


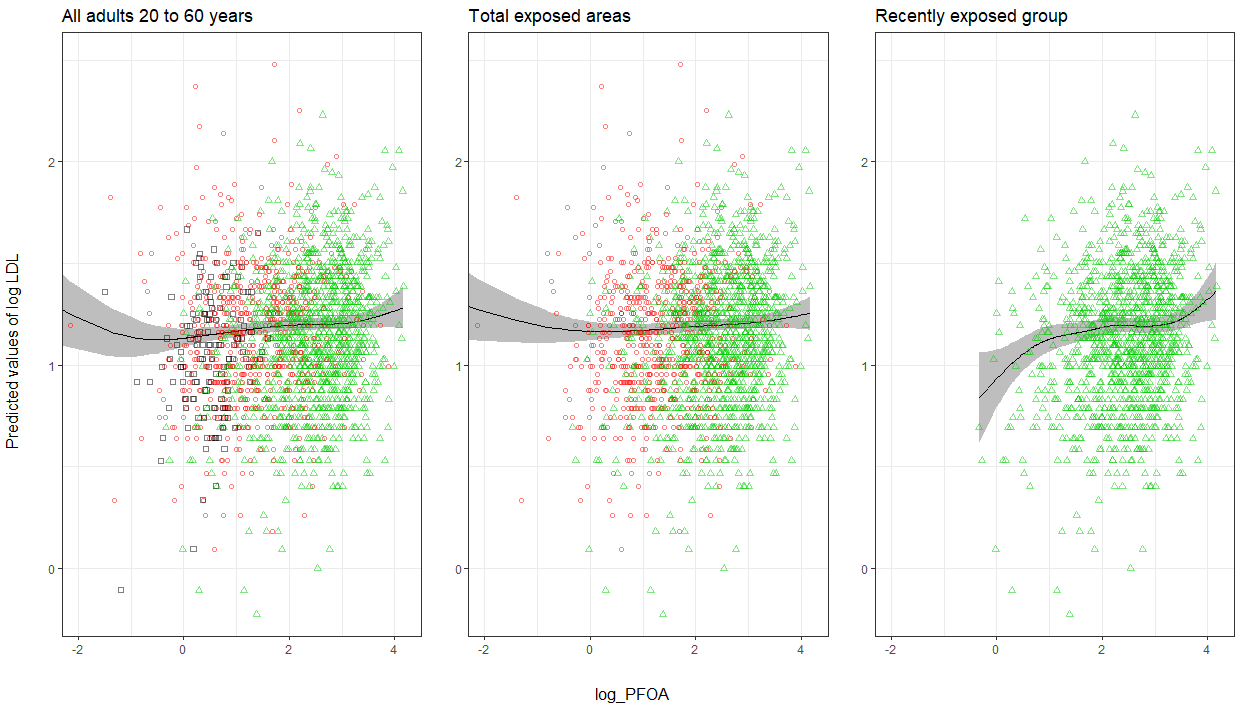


Figure S7. Smooth spline of ln-transformed LDL with 95% confidence bands, in Ronneby, Sweden, on ln-tranformed PFOA. The smooth spline function was adjusted by age, sex and BMI in quartiles. The points on the graph are the original data with black squares are the ones from the control group, the red circles are the ones from the non-recent or uncertain group and the green triangles are the ones from the recently exposed group.


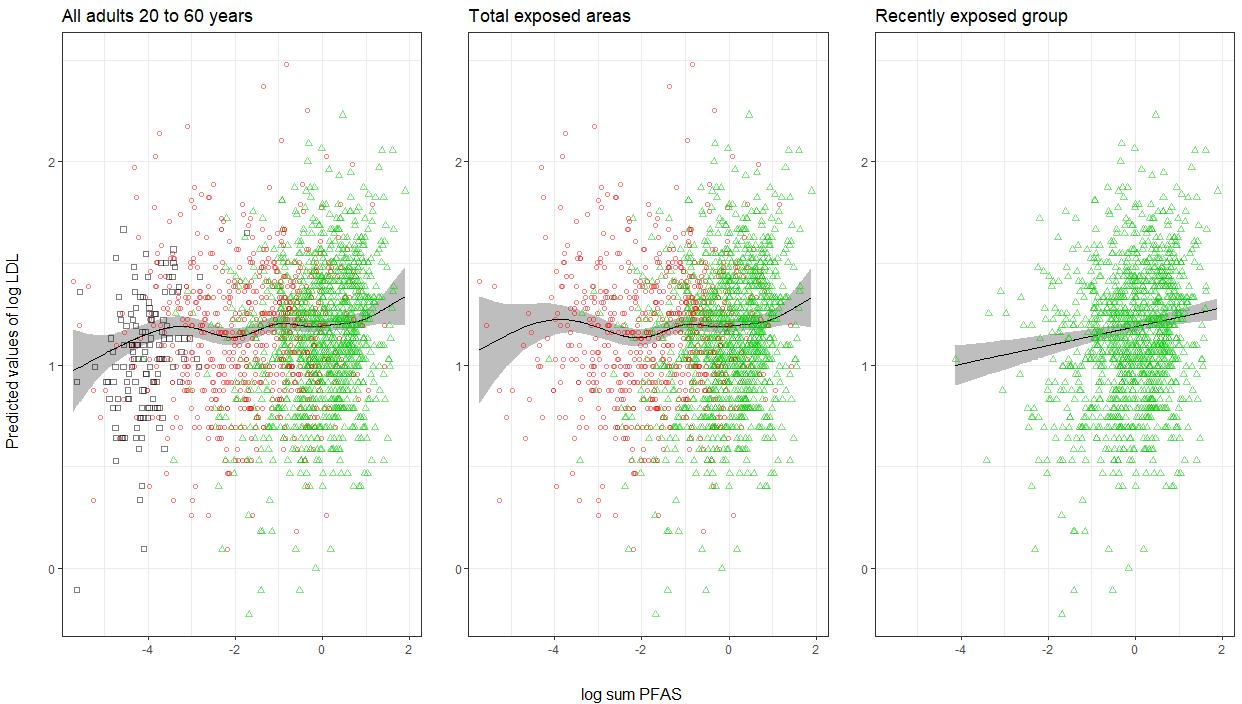


Figure S8. Smooth spline of ln-transformed LDL with 95% confidence bands, in Ronneby, Sweden, on ln-tranformed sum PFAS. The smooth spline function was adjusted by age, sex and BMI in quartiles. The points on the graph are the original data with black squares are the ones from the control group, the red circles are the ones from the non-recent or uncertain group and the green triangles are the ones from the recently exposed group.


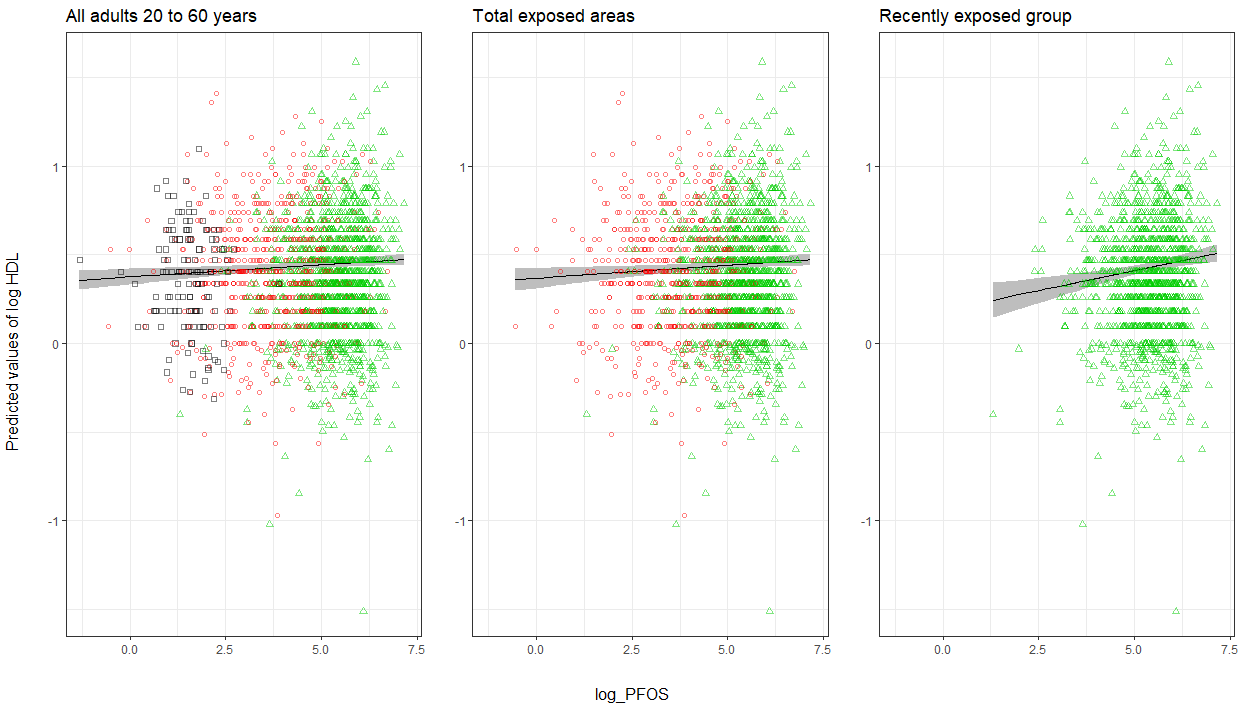


Figure S9. Smooth spline of ln-transformed HDL with 95% confidence bands, in Ronneby, Sweden, on ln-transformed PFOS. The smooth spline function was adjusted by age, sex and BMI in quartiles. The points on the graph are the original data with black squares are the ones from the control group, the red circles are the ones from the non-recent or uncertain group and the green triangles are the ones from the recently exposed group.


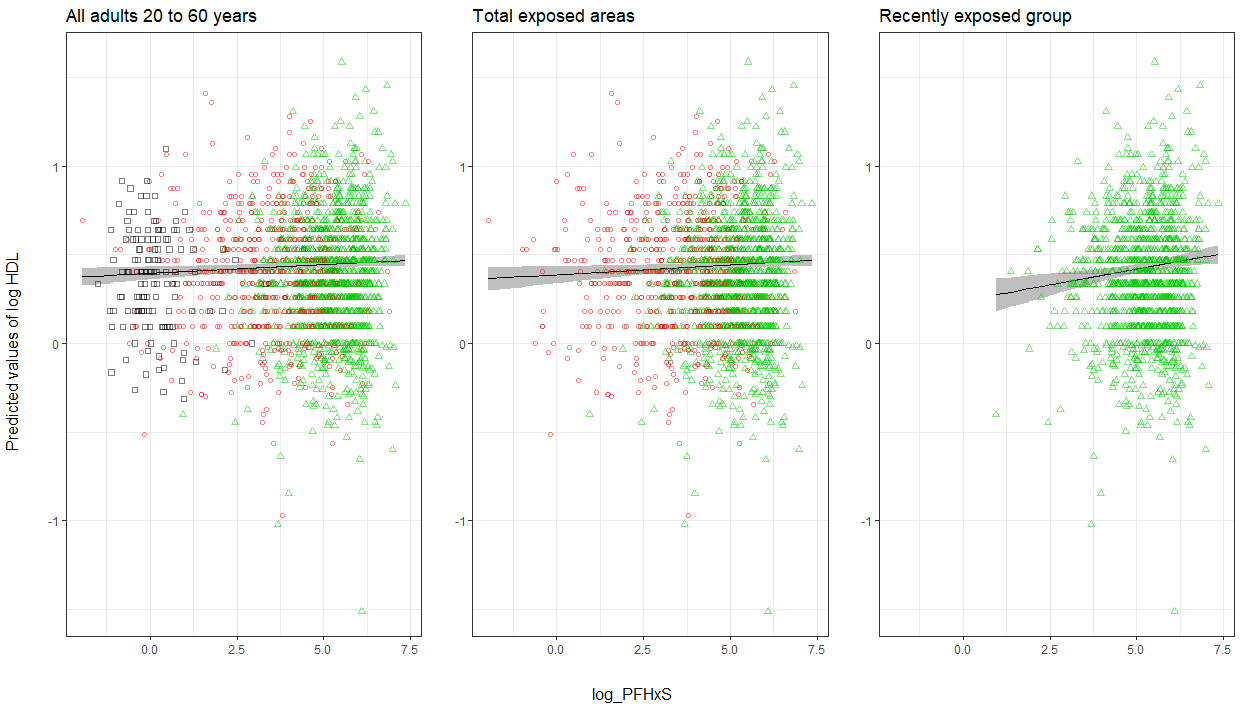


Figure S10. Smooth spline of ln-transformed HDL with 95% confidence bands, in Ronneby, Sweden, on ln-tranformed PFHxS. The smooth spline function was adjusted by age, sex and BMI in quartiles. The points on the graph are the original data with black squares are the ones from the control group, the red circles are the ones from the non-recent or uncertain group and the green triangles are the ones from the recently exposed group.


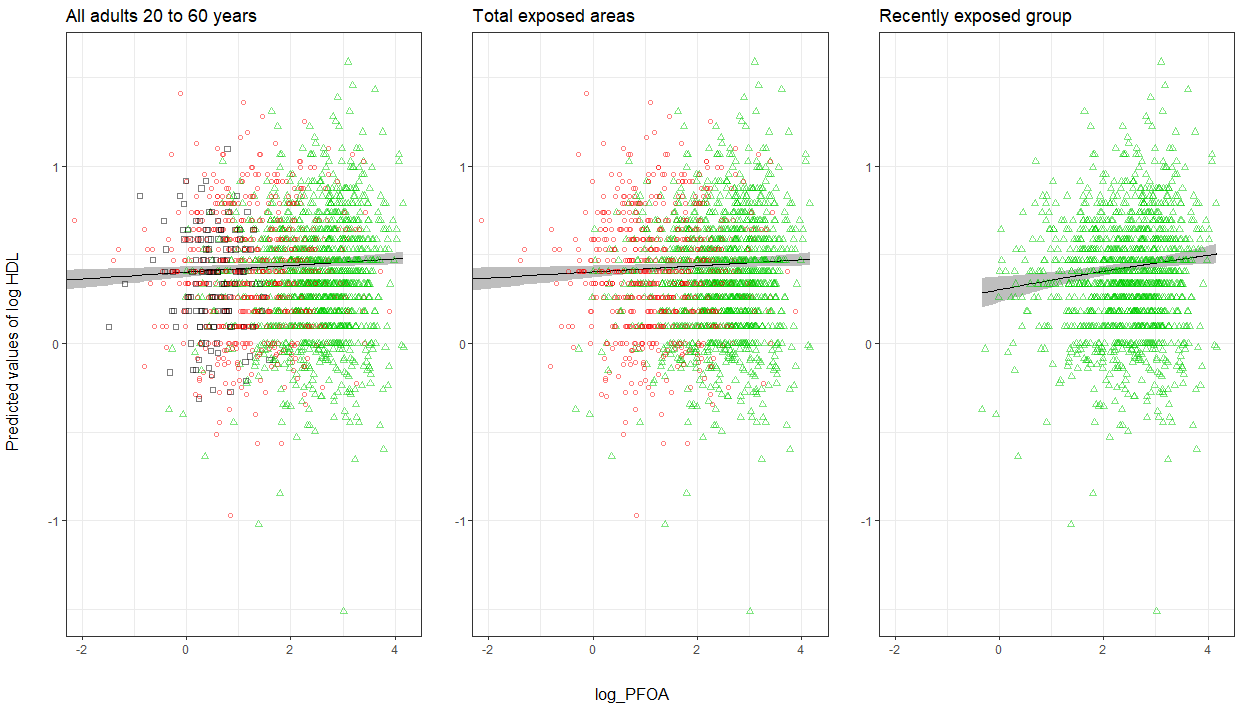


Figure S11. Smooth spline of ln-transformed HDL with 95% confidence bands, in Ronneby, Sweden, on ln-transformed PFOA. The smooth spline function was adjusted by age, sex and BMI in quartiles. The points on the graph are the original data with black squares are the ones from the control group, the red circles are the ones from the non-recent or uncertain group and the green triangles are the ones from the recently exposed group.


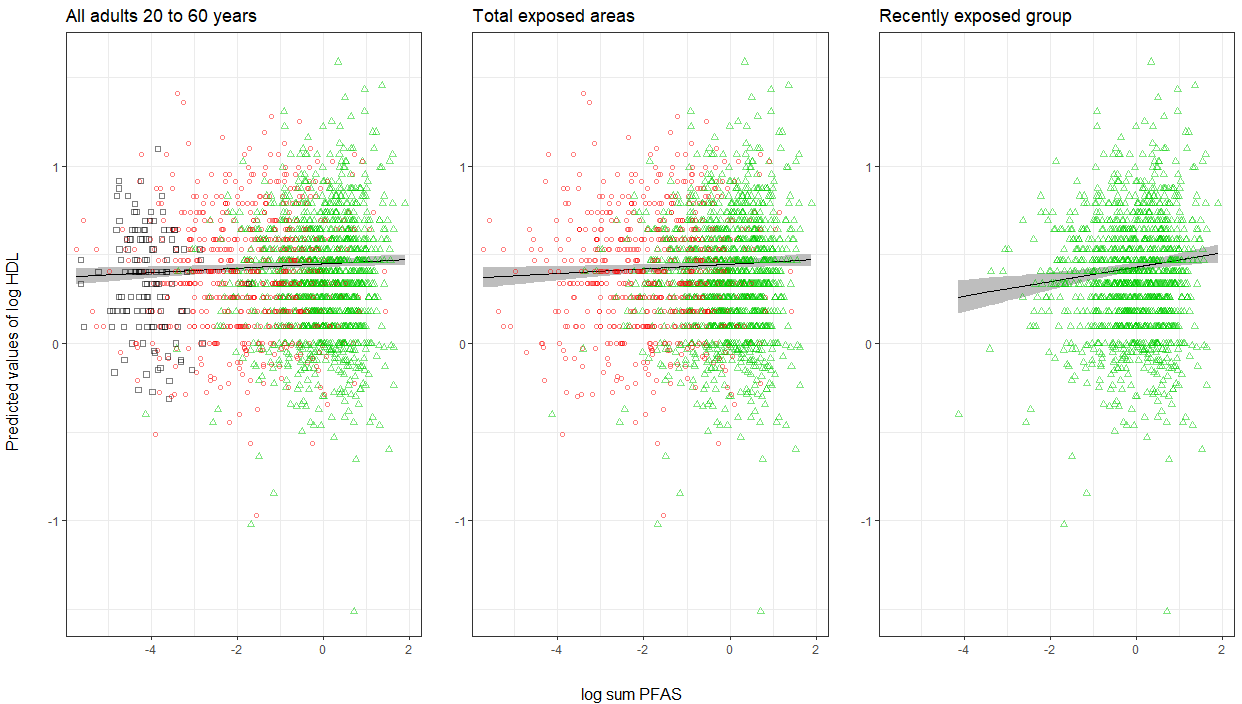


Figure S12. Smooth spline of ln-transformed HDL with 95% confidence bands, in Ronneby, Sweden, on ln-tranformed sum PFAS. The smooth spline function was adjusted by age, sex and BMI in quartiles. The points on the graph are the original data with black squares are the ones from the control group, the red circles are the ones from the non-recent or uncertain group and the green triangles are the ones from the recently exposed group


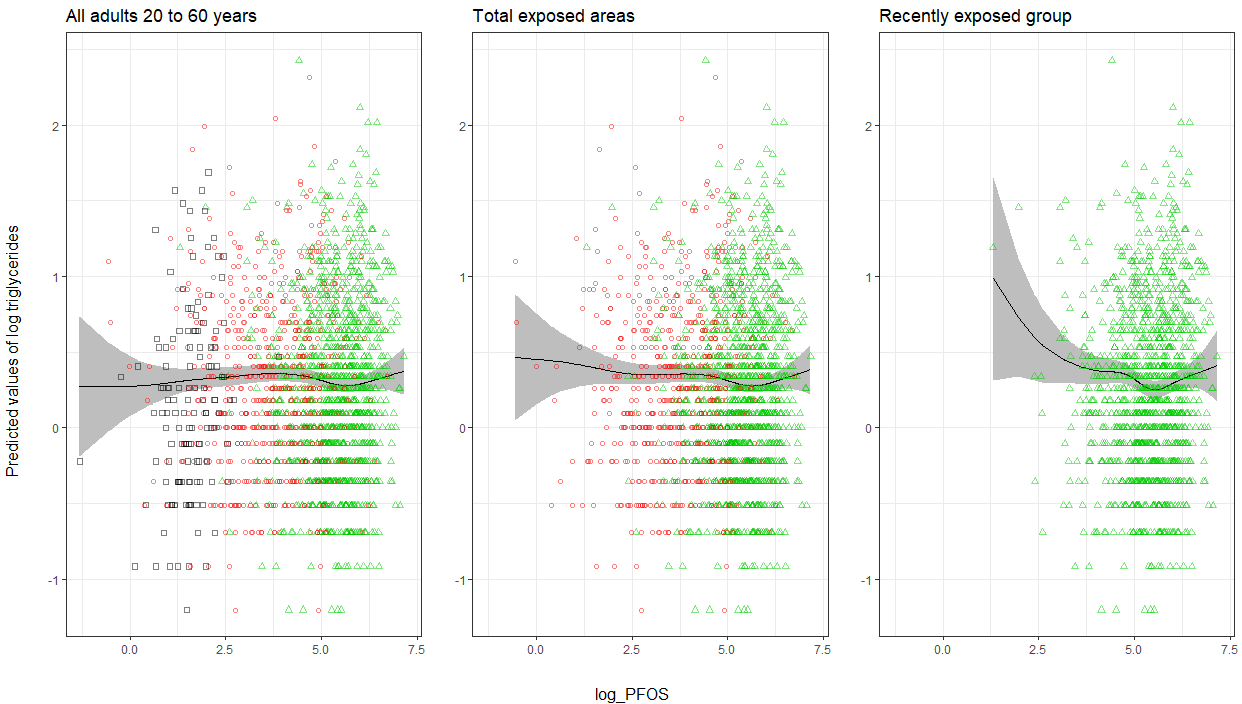


Figure S13. Smooth spline of ln-transformed triglycerides with 95% confidence bands, in Ronneby, Sweden, on ln-transformed PFOS. The smooth spline function was adjusted by age, sex and BMI in quartiles. The points on the graph are the original data with black squares are the ones from the control group, the red circles are the ones from the non-recent or uncertain group and the green triangles are the ones from the recently exposed group.


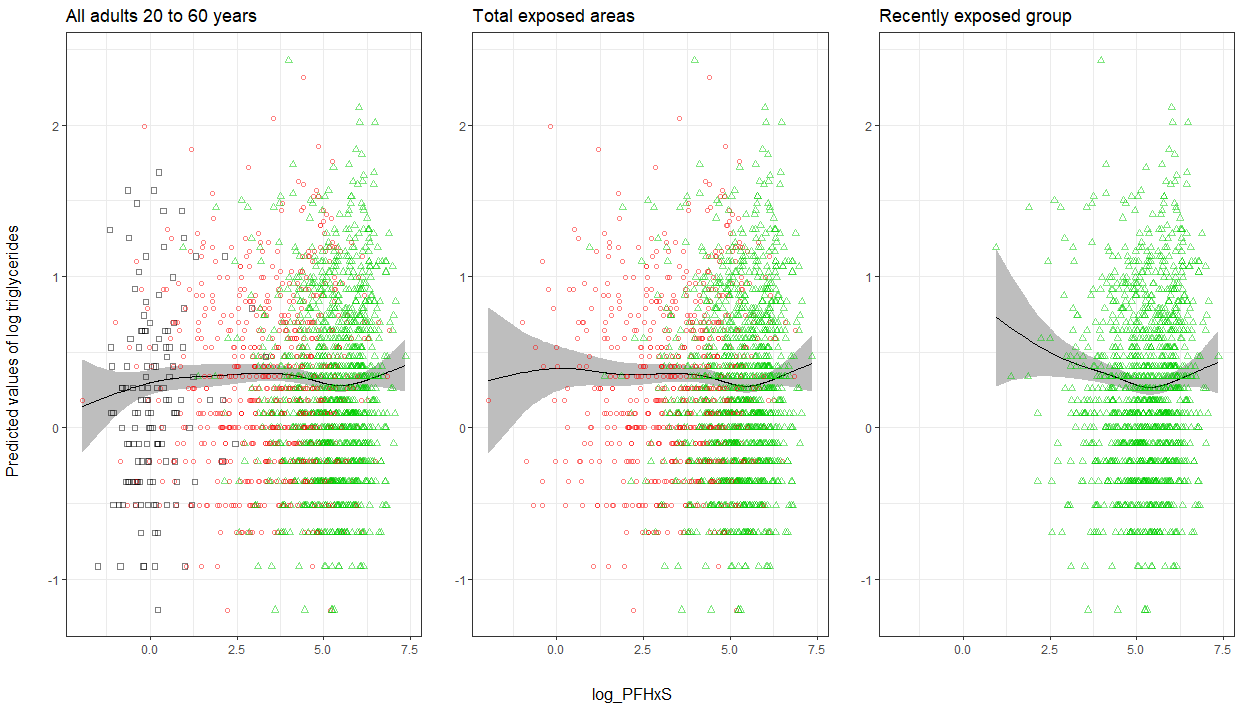


Figure S14. Smooth spline of ln-transformed triglycerides with 95% confidence bands, in Ronneby, Sweden, on ln-transformed PFHxS. The smooth spline function was adjusted by age, sex and BMI in quartiles. The points on the graph are the original data with black squares are the ones from the control group, the red circles are the ones from the non-recent or uncertain group and the green triangles are the ones from the recently exposed group.


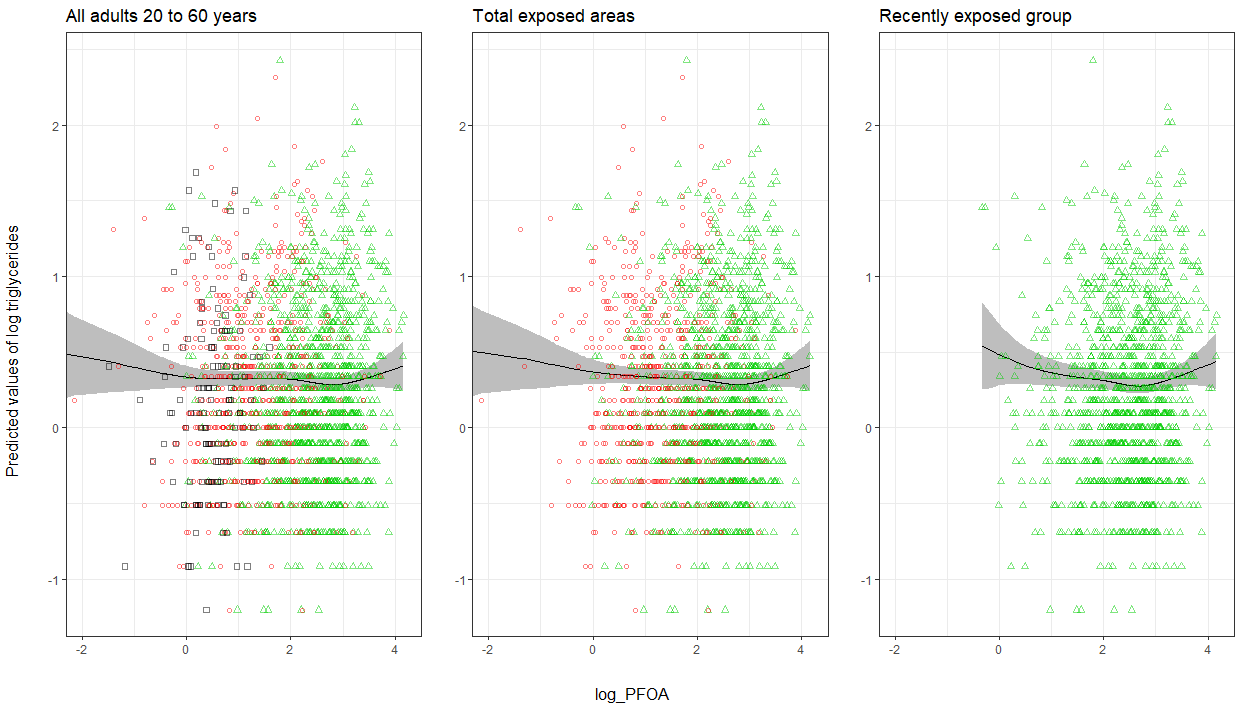


Figure S15. Smooth spline of ln-transformed triglycerides with 95% confidence bands, in Ronneby, Sweden, on ln-transformed PFOA. The smooth spline function was adjusted by age, sex and BMI in quartiles. The points on the graph are the original data with black squares are the ones from the control group, the red circles are the ones from the non-recent or uncertain group and the green triangles are the ones from the recently exposed group.


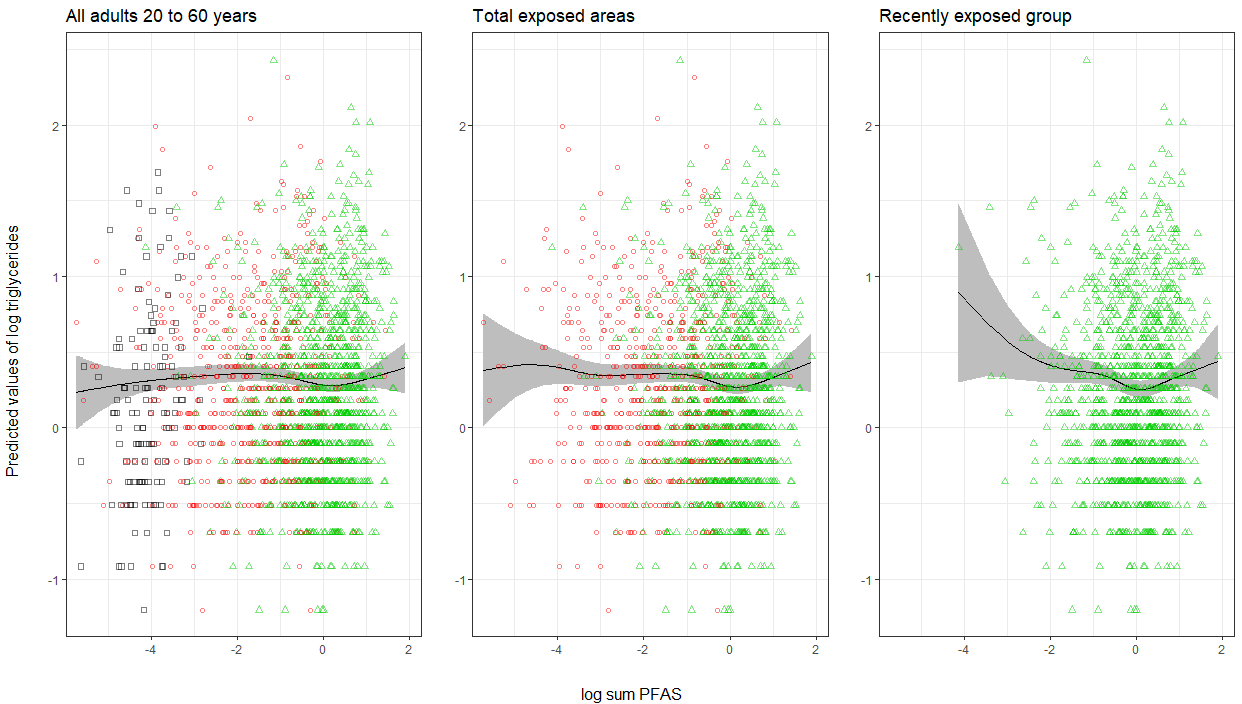


Figure S16. Smooth spline of ln-transformed triglycerides with 95% confidence bands, in Ronneby, Sweden, on ln-transformed sum PFAS. The smooth spline function was adjusted by age, sex and BMI in quartiles. The points on the graph are the original data with black squares are the ones from the control group, the red circles are the ones from the non-recent or uncertain group and the green triangles are the ones from the recently exposed group.


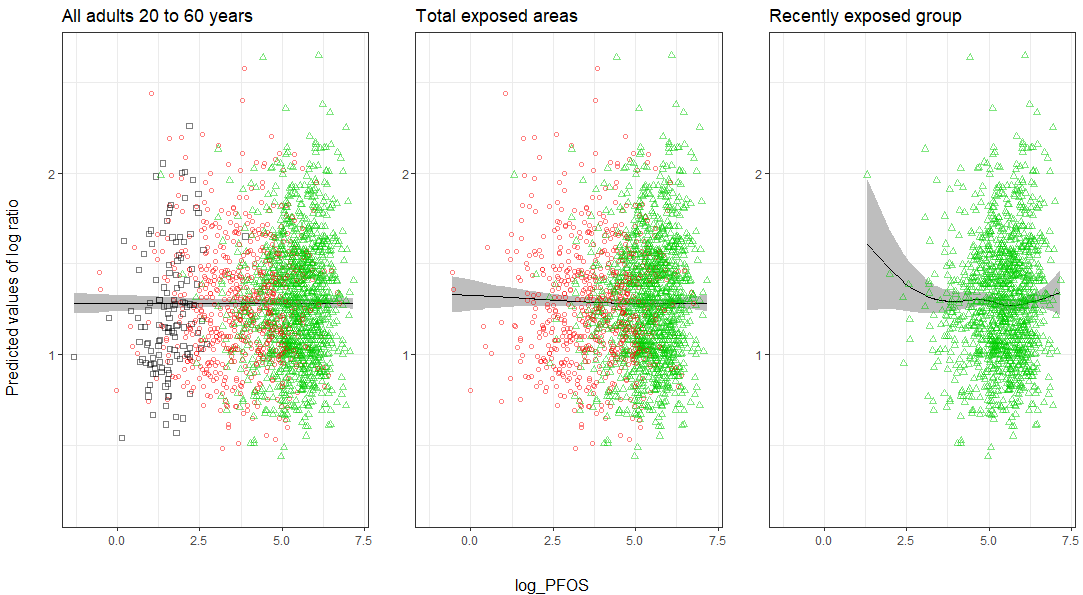


Figure S17. Smooth spline of ln-transformed total cholesterol/HDL ratio with 95% confidence bands, in Ronneby, Sweden, on ln-transformed PFOS. The smooth spline function was adjusted by age, sex and BMI in quartiles. The points on the graph are the original data with black squares are the ones from the control group, the red circles are the ones from the non-recent or uncertain group and the green triangles are the ones from the recently exposed group.


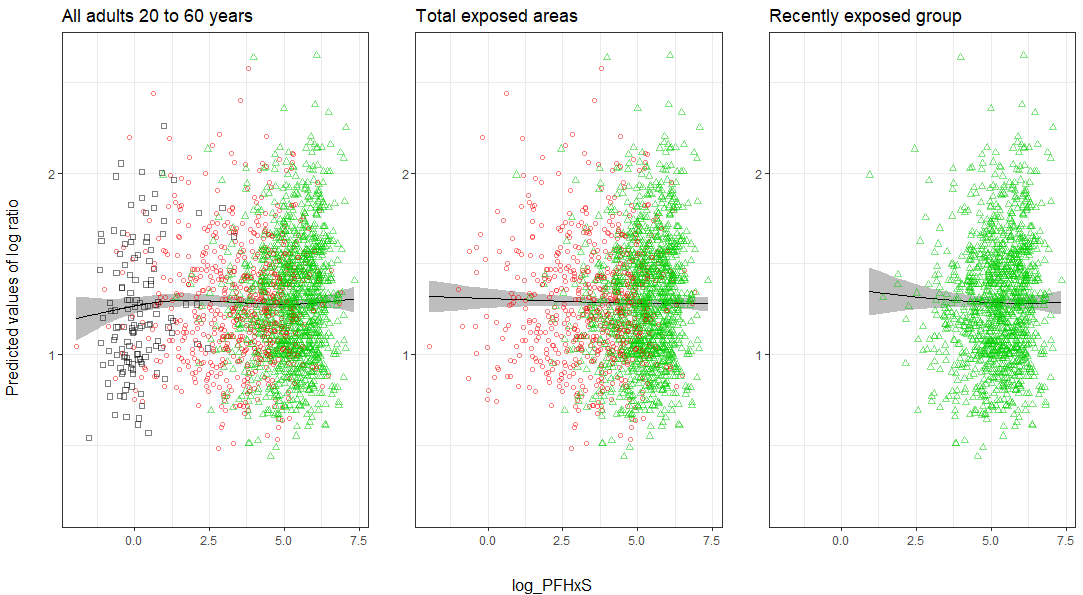


Figure S18. Smooth spline of ln-transformed total cholesterol/HDL ratio with 95% confidence bands, in Ronneby, Sweden, on ln-transformed PFHxS. The smooth spline function was adjusted by age, sex and BMI in quartiles. The points on the graph are the original data with black squares are the ones from the control group, the red circles are the ones from the non-recent or uncertain group and the green triangles are the ones from the recently exposed group.


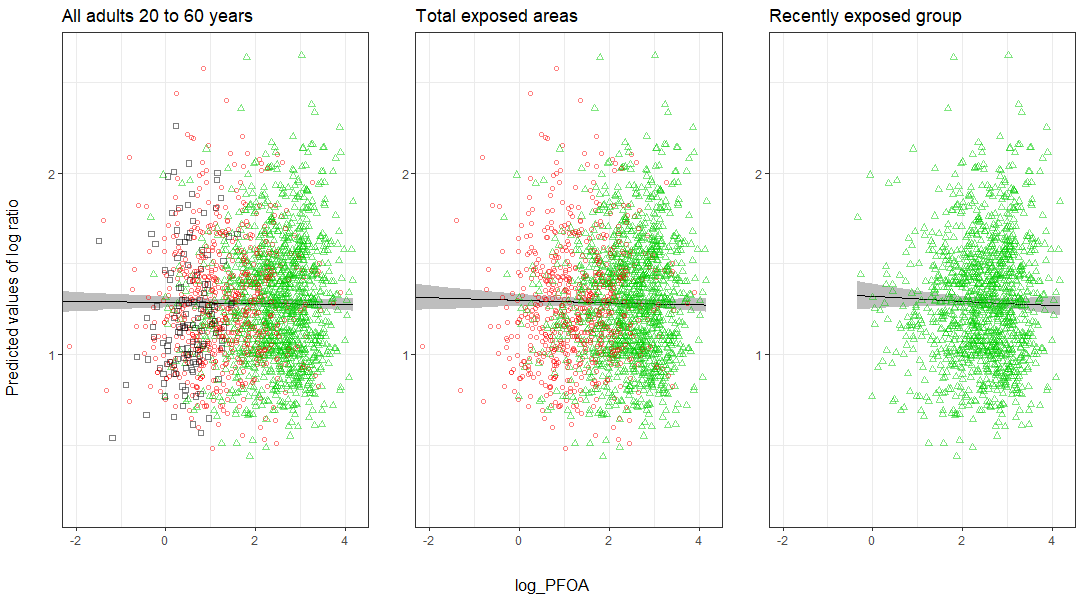


Figure S19. Smooth spline of ln-transformed total cholesterol/HDL ratio with 95% confidence bands, in Ronneby, Sweden, on ln-transformed PFOA. The smooth spline function was adjusted by age, sex and BMI in quartiles. The points on the graph are the original data with black squares are the ones from the control group, the red circles are the ones from the non-recent or uncertain group and the green triangles are the ones from the recently exposed group.


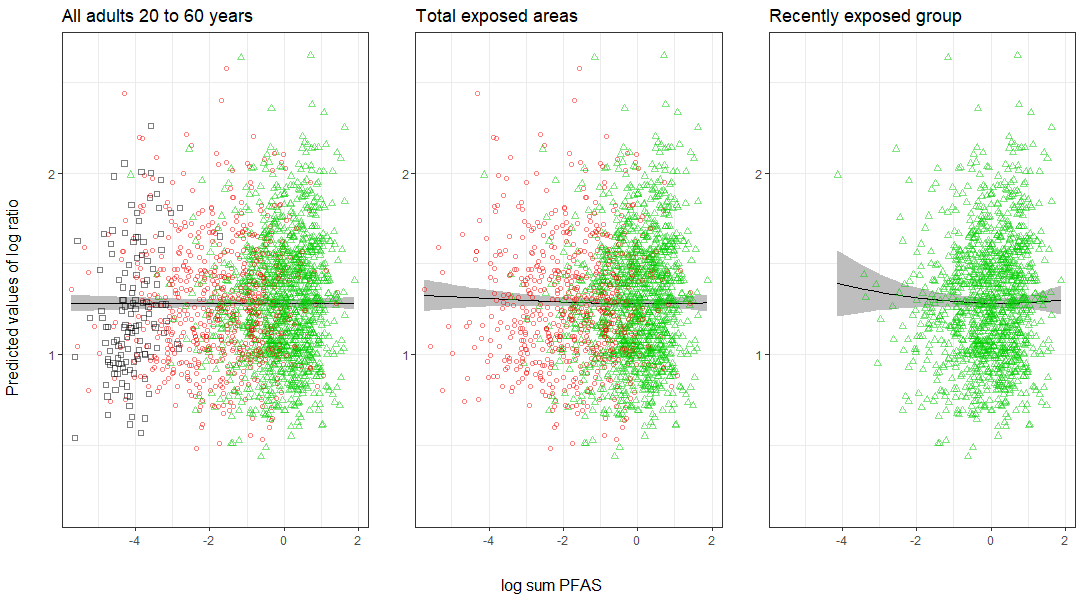


Figure S20. Smooth spline of ln-transformed total cholesterol/HDL ratio with 95% confidence bands, in Ronneby, Sweden, on ln-transformed sum PFAS. The smooth spline function was adjusted by age, sex and BMI in quartiles. The points on the graph are the original data with black squares are the ones from the control group, the red circles are the ones from the non-recent or uncertain group and the green triangles are the ones from the recently exposed group

***Supplementary Material 2: Chemical analysis of PFAS***

*Chemicals*

An overview of the PFAS analytes and internal standards with abbreviations is given in Table S17. All native and isotopically labelled standards were purchased from Wellington Laboratories (Guelph, Ontario, Canada) as diluted reference standards. Acetonitrile, ammonium acetate, and methanol were from Merck (Darmstadt, Germany). Water was from a Milli-Q Integral 5 system (Millipore, Billerica, MA, USA).

*Instrumentation*

Quantitative analysis was conducted using triple quadrupole linear ion trap mass spectrometers equipped with TurboIonSpray sources (QTRAP 5500; AB Sciex, Foster City, CA, USA) coupled to a liquid chromatography system (UFLCXR, Shimadzu Corporation, Kyoto, Japan; LC/MS/MS). Nitrogen was used as nebulizer, auxiliary, curtain and collision gas. The MS analyses were carried out using selected reaction monitoring (SRM) in negative ion mode. To establish the appropriate SRM conditions, standard solutions were infused into the MS for optimization and the SRM transitions giving the best signal to noise ratio were selected for analysis. The MRM conditions are shown in Table S18. All data acquisition was performed using Analyst 1.6.3 software and data processing was performed using Multiquant 2.1 (AB Sciex, Foster City, CA, USA).

*Sample preparation of serum samples from populations expected to have high exposure to PFAS*

Calibration standards, blanks and QC samples: Standard solutions were prepared by further dilution of diluted reference standards in water:acetonitrile (50:50). For the calibration standards a blank matrix of human serum was used and prepared in the same way as the samples, except for the addition of 25 µl diluted standard solutions. Three reference samples were prepared for quality control (QC1, QC2 and QC3) by pooling serum samples containing different levels of PFAS and additional spiking with PFAS standard solutions (Table S19). The three QC samples and four chemical blanks (water) and calibration standards were in each sample batch in a 96 well plate. The samples were prepared and analysed in duplicates and the mean of the two concentrations were used.

The samples were prepared in 96-well plates with 2 ml flat bottom glass vials (Biotech solutions, Vineland, NJ, USA). Aliquots of 25 µl sample and 75µl water were added with 10 µl glucuronidase and 10 µl 1M ammonium acetate buffer pH 6.5. The samples were digested at 37°C for 90 min. Thereafter 25 µl of a water:acetonitrile (50:50) and 25 µl of water:acetonitrile (50:50) solution containing isotopically labelled internal standards were added (Table S17). To precipitate the proteins, 200 µl of acetonitrile was added to all samples followed by vigorous shaking for 30 min. The samples were thereafter centrifuged at 2600g for 10 min. The supernatant (0.2mL) was transferred to a new 96-well plate with 0.5 mL conical glass vials (MicroLiter Analytical Supplies, Inc., Suwanee, GA, USA) for analysis and again centrifuged at 3000g for 10min before analysis.

An aliquot of 1µl of the supernatant were analysed on the LC/MS/MS. For the analysis a C_18_ column (2.1 mm i.d. x 50 mm, Genesis Lightn; Hichrom, Reading, UK) was used prior to the injector to filter the mobile phases. The analytical column was a 1.5 µm, 2.0 mm i.d. x 100 mm VisionHT C_18_ HL (Grace, Deerfield, IL, USA)

The mobile phases were A: 5mM ammonium acetate in water and B: methanol. The mobile phase was kept at 14% B for 1 min after injection. A gradient was then applied to 95% in 5 min B where it was kept for 1.5 min. The column was then conditioned at 14% B for 2 min. A diverter valve was used and the column effluent was diverted to the MS between 5.0 and 9.3 min. The flow rate was 0.28 mL/min and the column were maintained at 60°C. The analysis was performed in negative ion mode, the DP was 45V and the ion source temperature was at 630°C (Table S18).

*Sample preparation of serum samples from populations expected to have low exposure to PFAS*

Calibration standards, blanks and QC samples: Standard solutions were prepared by further dilution of diluted reference standards in water:acetonitrile (50:50). For the calibration standards a blank matrix of human serum was used and prepared in the same way as the samples, except for the addition of 25 µl diluted standard solutions. Two reference samples were prepared for quality control (QC4 and QC5) by pooling serum samples containing different levels (Table S20). The two QC samples and four chemical blanks (water) and calibration standards were in each sample batch in a 96 well plate. The samples were prepared and analysed in duplicates and the mean of the two concentrations were used.

The samples were prepared in 96-well plates with 2 ml flat bottom glass vials (Biotech solutions, Vineland, NJ, USA). Aliquots of 100 µl serum sample were added with 10 µl glucuronidase and 10 µl 1M ammonium acetate buffer pH 6.5. The samples were digested at 37°C for 90 min. Thereafter 25 µl of a water:acetonitrile (50:50) and 25 µl of water:acetonitrile (50:50) solution containing isotopically labelled internal standards were added (Table S17). To precipitate the proteins, 200 µl of acetonitrile was added to all samples followed by vigorous shaking for 30 min. The samples were thereafter centrifuged at 2600g for 10 min. The supernatant (0.2mL) was transferred to a new 96-well plate with 0.5 mL conical glass vials (MicroLiter Analytical Supplies, Inc., Suwanee, GA, USA) for analysis and again centrifuged at 3000g for 10min before analysis.

An aliquot of 4µl of the supernatant were analysed on the LC/MS/MS. For the analysis a C18 column (2.1 mm i.d. x 50 mm, Genesis Lightn; Hichrom, Reading, UK) was used prior to the injector to filter the mobile phases. The analytical column was a 1.5 µm, 2.0 mm i.d. x 100 mm VisionHT C18 HL (Grace, Deerfield, IL, USA)

The mobile phases were A: 5mM ammonium acetate in water and B: methanol. The mobile phase was kept at 14% B for 1 min after injection. A gradient was then applied to 95% in 5 min B where it was kept for 1.5 min. The column was then conditioned at 14% B for 2 min. A diverter valve was used and the column effluent was diverted to the MS between 5.0 and 9.3 min. The flow rate was 0.28 mL/min and the column were maintained at 60°C. The analysis was performed in negative ion mode, the declustering potential (DP) was 45V and the ion source temperature was at 630°C (Table S18).

*Quality*

The amount of PFAS in the method was determined as the total, non-isomer-specific compounds. The limits of detection (LOD), determined as the concentrations corresponding to three times the standard deviation of the responses in chemical blanks, are shown in Table S19. The results of the QC samples were used to calculate the between run precision of the method (Table S19 and S20), determined as the coefficient of variation (CV) of the quality control samples.

The analyses of PFOS and PFOA are part of a quality control programme between analytical laboratories coordinated by Professor Hans Drexler, Institute and Outpatient Clinic for Occupational, Social and Environmental Medicine, University of Erlangen-Nuremberg, Germany. The laboratory also participates in the ICI/EQUAS exercises for the analysis of PFOS, PFHxS, and PFOA and are approved by the HBM4EU project.

Table S17: Overview of the PFAS analytes and internal standards used

| Compound | Abbreviation | CAS |
| --- | --- | --- |
| Perfluoro-1-octanesulfonate | PFOS | 1763-23-1 |
| Perfluoro-1-hexanesulfonate | PFHxS | 355-46-4 |
| Perfluoro-n-octanoic acid | PFOA | 335-67-1 |
| Internal standards |  |  |
| Perfluoro-1-[1,2,3,4-^13^C_4_] octanesulfonate | ^13^C_4_-PFOS |  |
| Perfluoro-1-[^18^O_2_] hexanesulfonate | ^18^O_2_-PFHxS |  |
| Perfluoro-n-[1,2,3,4-^13^C_4_] octanoic acid | ^13^C_4_-PFOA |  |

Table S18: MRM parameters for the acquisition of PFAS and their internal standards.

|  | Transition | Collision energy (V) | PFAS-IS | Transition | Collision energy (V) |
| --- | --- | --- | --- | --- | --- |
| PFOS | 499/99 | -85 | ^13^C_4_-PFOS | 503/80 | -110 |
| PFHxS | 399/80 | -80 | ^18^O_2_-PFHxS | 403/103 | -80 |
| PFOA | 413/169 | -25 | ^13^C_4_-PFOA | 417/372 | -25 |

Table S19. Limit of detection (LOD) and the concentrations and coefficient of variation in the quality control (QC) samples in serum samples from populations expected to have high exposure to PFAS were prepared using aliquots of 25 μl serum added with 75 μl of water and 1µl of the samples were analyzed using LC/MS/MS.

|  | LOD (ng/ml) | QC1 (ng/ml) | between-run precision CV( %) | QC2 (ng/ml) | between-run precision CV( %) | QC3 (ng/ml) | between-run precision CV( %) |
| --- | --- | --- | --- | --- | --- | --- | --- |
|  |  |  |  |  |  |  |  |
| PFOS | 0.5 | 6 | 12 | 10 | 10 | 100 | 5 |
| PFHxS | 0.5 | 2 | 19 | 4 | 12 | 99 | 6 |
| PFOA | 0.4 | 3 | 11 | 4 | 10 | 103 | 8 |

Table S20. Limit of detection (LOD) and the concentrations and coefficient of variation in the quality control (QC) samples in serum samples from populations expected to have lower exposure to PFAS were prepared using aliquots of 100 μl serum were used and 4µl of the samples were analyzed using LC/MS/MS.

|  | LOD (ng/ml) | QC 4 (ng/ml) | between-run precision CV( %) | QC 5 (ng/ml) | between-run precision CV( %) |
| --- | --- | --- | --- | --- | --- |
|  |  |  |  |  |  |
| PFOS | 0.1 | 12 | 6 | 13 | 4 |
| PFHxS | 0.1 | 2 | 7 | 3 | 7 |
| PFOA | 0.1 | 3 | 10 | 5 | 10 |
